# Genome Mining-Driven Isolation of New Gromomycins and Insights into Their Mode of Action

**DOI:** 10.1021/acschembio.5c00821

**Published:** 2026-03-05

**Authors:** Dmytro Bratiichuk, Franziska Fries, Marc Stierhof, Leon Morguet, Josef Zapp, Mathias Müsken, Yuriy Rebets, Maksym Myronovskyi, Rolf Müller, Jennifer Herrmann, Andriy Luzhetskyy

**Affiliations:** † Department of Pharmaceutical Biotechnology, 9379 Saarland University, Bld. C2.3, 66123 Saarbrücken, Germany; ‡ Helmholtz Institute for Pharmaceutical Research Saarland (HIPS), Helmholtz Centre for Infection Research (HZI), 9379 Saarland University, 66123 Saarbrücken, Germany; § German Center for Infection Research (DZIF), Partner Site Hannover-Braunschweig, 38124 Braunschweig, Germany; ∥ 443745 Helmholtz Centre for Infection Research (HZI), 38124 Braunschweig, Germany; ⊥ German-Ukrainian Core of Excellence in Natural Products Research (CENtR), Zelena st. 20, 79005 Lviv, Ukraine

## Abstract

The growing threat of multidrug-resistant bacterial infections highlights the urgent need for antibiotics with novel mechanisms of action. Gromomycins, a newly identified class of triterpene antibiotics, exhibit potent activity against Gram-positive bacteria, including drug-resistant species, through a previously uncharacterized mode of action. Here, we report the discovery of a gromomycin-like biosynthetic gene cluster in the *Actinoplanes* genus through a genome mining approach, leading to the isolation and characterization of new bioactive derivatives that overcome resistance to clinically used drugs in vancomycin-resistant enterococci. Mechanistic studies revealed that gromomycins induce rapid potassium ion leakage and depolarization of the bacterial membrane, resulting in bactericidal activity against *Staphylococcus aureus*. Gromomycins disrupt the integrity of the cytoplasmic membrane, as evidenced by large pore formation, leakage of intracellular contents, and subsequent cell lysis. Supplementation with membrane lipids and fatty acids neutralized their antibacterial activity, suggesting a direct membrane-targeting mechanism, further supported by the inability to raise gromomycin resistance and their toxic effects on eukaryotic cells. Collectively, these findings deepen our understanding of gromomycin activity and demonstrate the utility of genome mining to uncover structurally novel and biologically active natural products.

## Graphical abstract

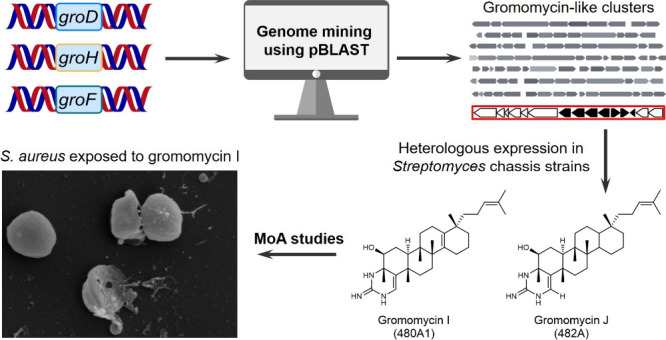

## Introduction

Actinomycetota (previously known as Actinobacteria) have long been recognized as a key provider of many natural products (NPs) throughout the years.^1–4^ *Streptomyces*, a highly characterized genus of actinomycetes, is viewed as one of the most essential industrial bacteria due to its significant potential for generating secondary metabolites, including antibiotics, immunosuppressants, and anticancer agents.^5–7^ However, ongoing studies of *Streptomyces* have made it increasingly challenging to discover new compounds that exhibit strong antibacterial properties from this genus. On the contrary, many non-*Streptomycetes* belonging to genera, such as *Actinoplanes*, *Micromonospora*, *Saccharopolyspora*, *Nocardia*, *Actinomadura*, *Amycolatopsis*, and *Streptoverticillium* have become potential resources for antibiotic discovery, generating distinct compounds with significant antibacterial activity.^8–13^

The early 2000s marked the emergence of microbial genome mining as a strategy to enhance drug discovery, based on the insight that newly sequenced actinomycete genomes encode a much larger number of secondary metabolite biosynthetic gene clusters (BGCs) than was anticipated from established secondary metabolomes.^14–16^ With the development of rapid and affordable sequencing technologies, the understanding of this phenomenon has further strengthened.^17,18^ The identification and study of new classes of bacterial NPs and the development of different bioinformatics tools for BGCs identification, such as antiSMASH, PRISM, ClusterFinder, PKMiner, SBSPKS, RiPPER, etc., have significantly sped up the discovery of new compounds from bacterial sources.^19–27^ At the same time, the majority of these instruments are primarily based on existing knowledge about the biosynthetic principles of known classes of secondary metabolites. This causes certain limitations that these tools are facing; they cannot recognize new types of biosynthesis and thus identify new classes of bacterial NPs.^28,29^

Recently, we reported the structure, activity, and biosynthetic pathway of a new family of bacterial natural products named gromomycins from *Streptomyces* sp. Je 1–332 (gromomycins A, B) and *Streptomyces flavoviridis* (gromomycins E, F) (Figure 1A).^30^ Gromomycins are pentacyclic triterpenes with a cyclic guanidino group forming the fifth six-membered ring. They represent a new type of biosynthetic logic of secondary metabolism, being the first bacterial triterpenes synthesized independently of the squalene pathway and exhibiting an unprecedented cyclization route that utilizes a hexaprenylguanidine linear precursor. Gromomycins E and F from *S. flavoviridis* differ from A and B derivatives by the methylation of the side chain, which is performed by an additional gene encoding a protein with a methyltransferase domain in the *S. flavoviridis* gromomycin-like BGC (*gro*BGC).^30^ On the other hand, these compounds have a pronounced antimicrobial activity against methicillin-resistant, vancomycin-intermediate (VISA), resistant, daptomycin-resistant *Staphylococcus aureus* strains, as well as a substantial activity against *Mycobacterium tuberculosis* and *Acinetobacter baumannii*.^30^ This activity seems to be strongly dependent on variations in the structure of the side chain of the compound, prompting the search for new gromomycin derivatives. At the same time, the mode of action and, thus, the cellular target(s) of these compounds remained unclear.

**Figure 1:**
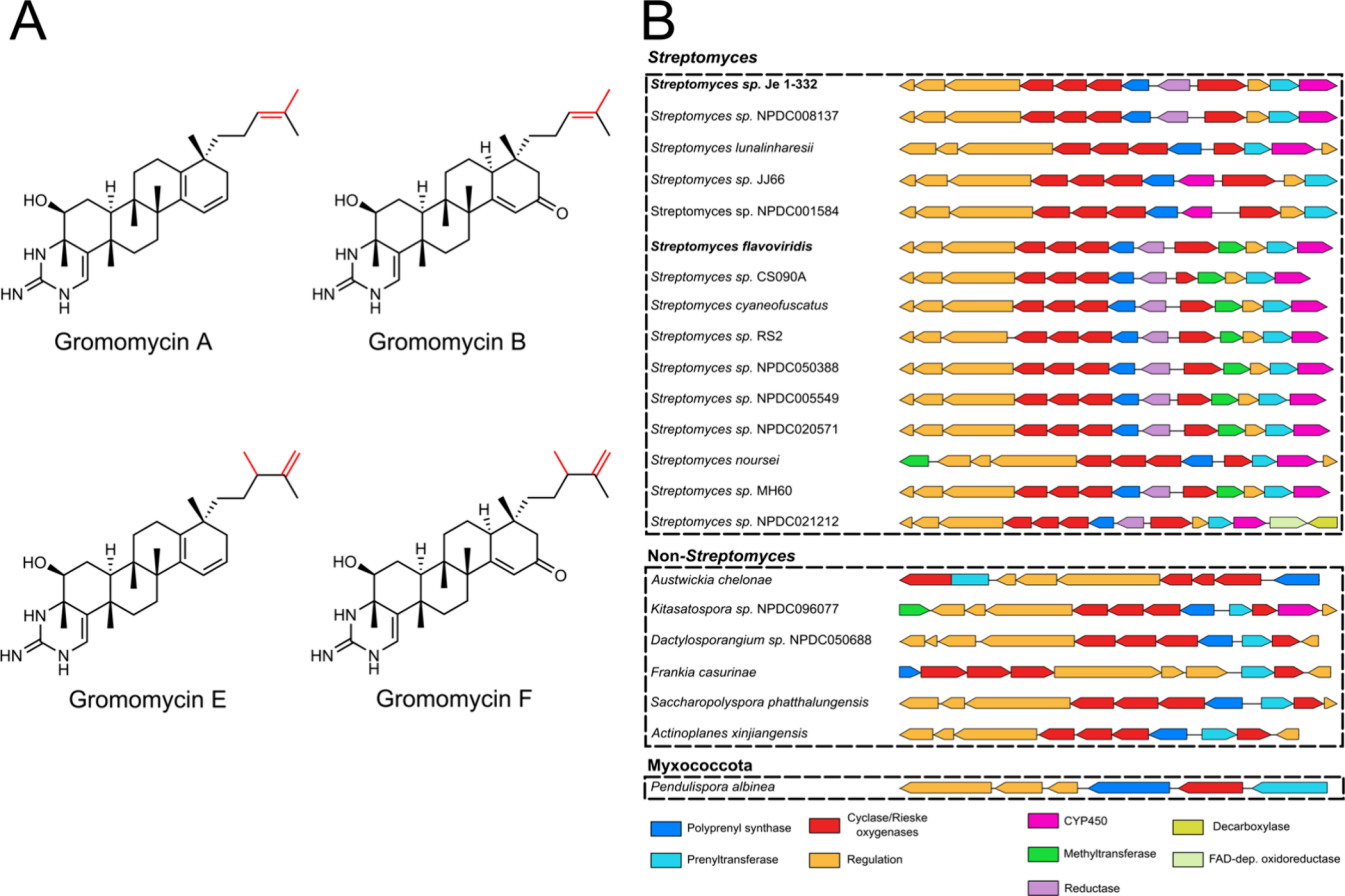
(A) Structures of gromomycins A and B and E and F (methylated). (B) Gromomycin BGC and its homologues.

Here, we report the identification of a novel *gro*BGC from the genus *Actinoplanes*, which resulted in the isolation and characterization of new biologically active gromomycin derivatives with potent activity against vancomycin-resistant *Enterococcus faecium* isolates. The use of a new producer strain with a higher yield of compounds than that of *Streptomyces* sp. Je 1–332 resulted in better access to these antibiotics and thus allowed for mode-of-action studies. In-depth mechanistic profiling revealed that gromomycins target the cytoplasmic membrane, which underlies their rapid bactericidal effect, but also accounts for their off-target activity toward eukaryotic cells.

## Results and Discussion

### 1 Identification of New Gromomycin-Like BGCs in *Actinoplanes*

Gromomycins are a new class of bacterial natural products with a distinct biosynthetic pathway that previously could not be recognized by pattern-based BGC prediction tools. Elucidation of the gromomycin biosynthetic pathway laid the foundation for a genome mining approach, which was used to explore the distribution of *gro*BGCs and to identify new derivatives.^30^ The core part of the gromomycin BGC is represented by three genes: farnesyl diphosphate synthase (*groD*), prenyltransferase (*groH*), and cyclase (*groF*) (Figure 1B). We used the nucleotide sequences of these genes as probes to search for related BGCs within the genomes of Actinomycetota deposited in the NCBI GenBank database. Since the enzymes involved in gromomycin assembly are highly abundant within bacterial genomes and are involved in different processes, the ultimate search criteria were the proximity of all three genes.

The search revealed that a number of *Streptomyces* and non-*Streptomyces* Actinomycetota harbor *gro*BGC homologues within their genomes. The selected groBGCs are represented in Figure 1B. Further examination showed that the strains *Streptomyces lunalinharesii*, *Streptomyces* sp. JJ66, *Streptomyces* sp. NPDC008137, *Streptomyces* sp. NPDC001584 all possess a gromomycin BGC almost identical to that of *Streptomyces* sp. Je 1–332. At the same time, six strains (*Streptomyces* sp. CS090A, *Streptomyces cyaneofuscatus*, and *Streptomyces* sp. RS2, *Streptomyces* sp. NPDC050388, *Streptomyces* sp. NPDC005549, and *Streptomyces* sp. NPDC020571) include an additional gene encoding a protein with a methyltransferase domain. Additionally, *Streptomyces noursei* harbors a *gro*BGC with an additional class I SAM-dependent methyltransferase, whereas the cluster from *Streptomyces* sp. MH60 contains a gene annotated as a 27-*O*-demethylrifamycin SV methyltransferase. These seven *gro*BGCs encompass a set of genes similar to those previously found in *S. flavoviridis*, which is known to produce methylated gromomycins (Figure 1A).^30^ Notably, the strain *Streptomyces* sp. NPDC021212 was found to contain a *gro*BGC carrying two additional genes, encoding a putative FAD-dependent oxidoreductase and a uroporphyrinogen decarboxylase. These enzymes might be involved in new tailoring modifications during gromomycin biosynthesis and the generation of novel derivatives.

Meanwhile, the groBGCs were found within the genomes of rare *Actinomycetota* strains, such as *Austwickia chelonae* and *Kitasatospora* sp. NPDC096077, *Dactylosporangium* sp. NPDC050688, *Frankia casurinae*, *Saccharopolyspora phatthalungensis*, and *Actinoplanes xinjiangensis*. A detailed analysis revealed that the *Kitasatospora* sp. NPDC096077 cluster is virtually identical to the *S. flavoviridis* gromomycin BGC, while the clusters of the other five strains differ by the absence of several biosynthetic genes. In particular, the BGC from the *A. xinjiangensis* strain lacks groI and groE, which encode CYP450 monooxygenase and reductase tailoring enzymes involved in the incorporation of a keto group at C-17 of gromomycins and its subsequent reduction to a hydroxyl group, which, in turn, degrades, generating gromomycin A.^30^

It is noteworthy that *gro*BGCs were identified within the genomes of phyla other than Actinomycetota, including Myxococcota species, and particularly the *Pendulispora albinea* strain. Its *gro*BGC contains only homologues of the core genes *groD*, *groH*, and *groF*, while lacking genes encoding other enzymes, suggesting the potential to produce novel derivatives.

Additionally, the prevalence of *gro*BGCs in bacterial genomes was further examined using the recently developed bioinformatics tool CluSeek.^31^ Unlike methods that rely on predefined cluster types or reference libraries, CluSeek enables mining any gene neighborhoods containing colocalized homologues of user-specified genes. Using *groD*, *groF*, and *groH* as probes, CluSeek analysis confirmed the widespread distribution of *gro*BGCs in both Actinomycetota and Myxococcota (Figure S20). Furthermore, several cyanobacterial genomes were found to harbor *gro*BGCs, indicating the potential for the biosynthesis of new analogues.

### 2 Heterologous Expression of *A. xingiangensis gro*BGC and Isolation of New Derivatives

A genome library of the *Actinoplanes xinjiangensis* DSM 45184 strain was constructed, using the ϕ*C31*-based integrative cosmid vector cos15AAmInt. The library was end-sequenced and mapped to the genome of the *A. xinjiangensis* DSM 45184 (GenBank ref no. GCF_003148685.1). The cosmid clone P19–C01 was found to carry a 31.7 kb fragment of the *A. xinjiangensis* chromosome with th*gro*BGC. The cosmid was additionally confirmed by PCR. The P19–C01 cosmid was introduced into *Streptomyces albus* Del14 (now *Streptomyces albidoflavus* Del14) and *Streptomyces lividans* ΔYA9 strains.^32,33^ The obtained transconjugants were cultured in SG, DNPM, or GYM media, and metabolites were extracted with ethyl acetate. The high-resolution LC-MS analysis of extracts of both *S. albus* Del14 and *S. lividans* ΔYA9 strains, containing the P19–C01 cosmid, revealed several distinct peaks (Figure 2A). The mass spectral analysis showed molecular ions with *m*/*z* of 480.39 [M + H]^+^ (480A1), 480.39 [M + H]^+^ (480A2), and 482.41 [M + H]^+^ (482A) (Figure S19). To determine the structures of the identified compounds, strain *S. albus* Del14-P19-C01 was grown in 10 L of SG medium, and metabolites were extracted from the supernatant with ethyl acetate. Compounds 480A1, 480A2, and 482A were purified, and their structures were determined by NMR (Figures 2B, S1–S16, and Table S1).

**Figure 2:**
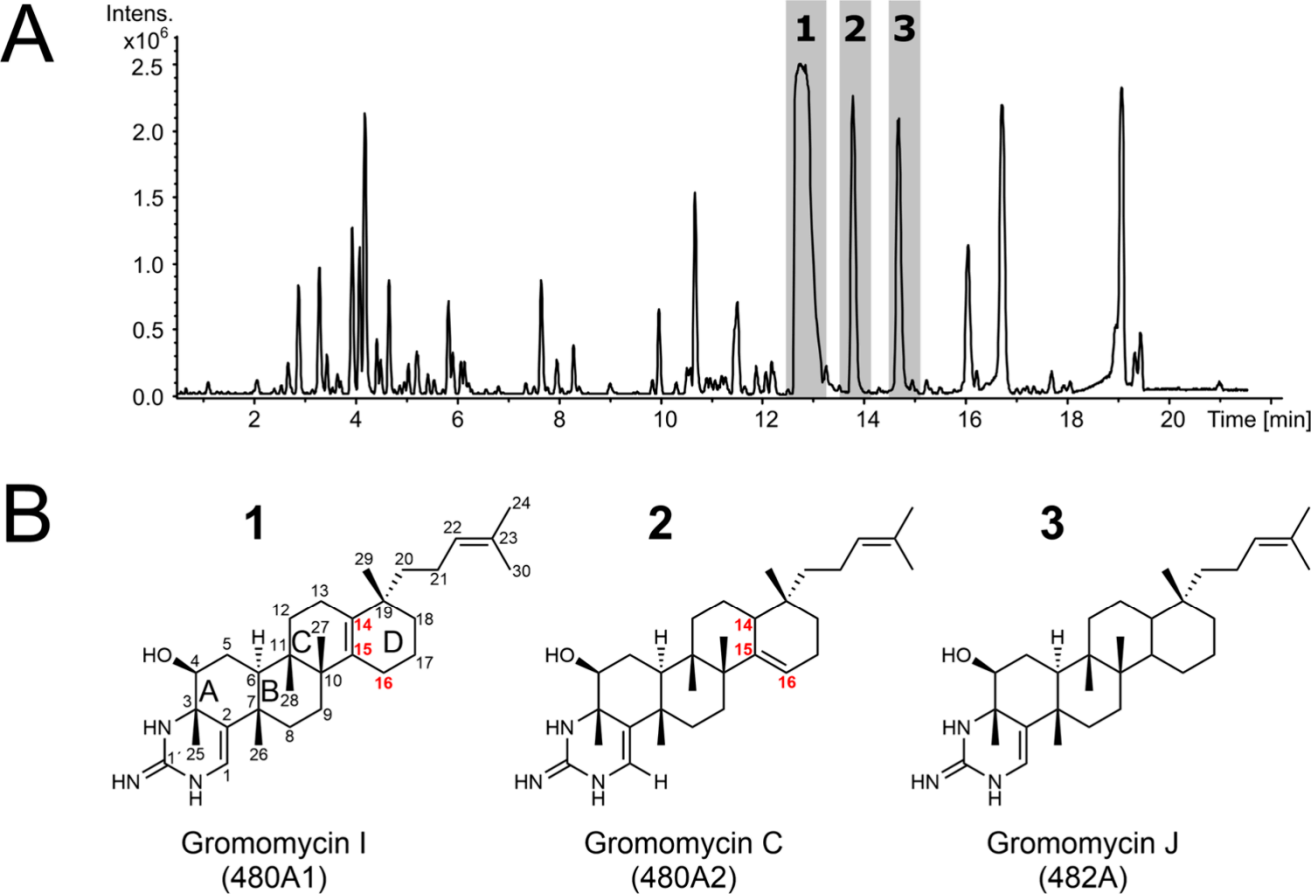
(A) Heterologous expression of cosmid P19–C01 with *gro*BGC into *S. albus* Del14. Peaks induced by clusters are marked by numbers: (1) Gromomycin I (480A1), (2) Gromomycin C (480A2), and (3) Gromomycin J (482A). (B) Structures of gromomycins I, C, and J.

Compounds 480A1 (**1**) and 480A2 (**2**) (Figure 2) share the same molecular formula, C_31_H_49_N_3_O, identical to that of gromomycin C, which is a key intermediate in gromomycin biosynthesis.^30^ Comparison of the ^1^H and ^13^C NMR data of 480A2 with those of gromomycin C confirmed that the two compounds are structurally identical. In contrast, the same mass but a different retention time of the compound 480A1 indicates that it is a structural isomer of gromomycin C. Full assignment of the structure using HMBC correlations from CH_3_-29 to C-14 and from CH_3_-27 to C-15 revealed that the double bond in ring D, originally located at C-15/C-16 in gromomycin C, had shifted to C-14/C-15 in 480A1 (Figures 2B, S1, S6, and S11). This new derivative was named gromomycin I. Compound 482A (**3**) has a calculated molecular formula of C_31_H_51_N_3_O, indicating the loss of one double bond. This was supported by the COSY and HMBC correlations, which showed that ring D is fully saturated (Figure 2B). The origin of this derivative, designated as gromomycin J, remains unclear. Since there are no respective genes on the P19–C01 cosmid that could explain the reduction of the C=C bond of gromomycin, we anticipate that it may be a shunt product arising during the cyclization of the linear precursor.

Based on the previously postulated minimal gene cluster for gromomycin synthesis, we have bioinformatically identified the borders of *A. xinjiangensis* *groBGC* spanning from gene 6 to gene 15 (Figure 3 and Table 1). Within this range, we propose that genes 9–14 are structural (designated as *groA* to *groF*), whereas genes 6 to 8 and 15 are likely to be regulatory genes (designated as *gro6* to *gro8* and *gro15*). The right boundary of the *A. xinjiangensis* *groBGC* is defined by gene *gro15* encoding a protein-tyrosine phosphatase, and shares homology with gene *gro3* from *Streptomyces* sp. Je 1–332. Similarly, *gro6*, located at the left boundary of the *A. xinjiangensis* groBGC, corresponds to *gro4* (encoding a lysylphosphatidylglycerol synthase transmembrane domain-containing protein) in the *Streptomyces* sp. Je 1–332. The deletion of genes *gro3* and *gro4* from *Streptomyces* sp. Je 1–332 *gro*BGC was associated with a substantial decrease in gromomycin levels. Furthermore, homologues of *gro6*, *gro7*, *gro8*, and *gro15* are conserved across other *gro*BGCs described in this study (Figure 1B), supporting the notion that these genes represent common and functionally important components of this type of BGC.

**Figure 3:**
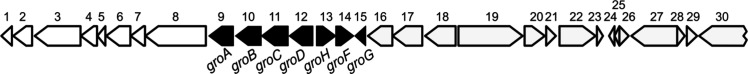
Diagram of the DNA segment containing the *A. xinjiangensis* *gro*BGC is depicted in black.

**Table 1:** Proposed Function of Genes in *A. xingiangensis gro*BGC

**gene**	**proposed function**
*9 (groA)*	Rieske-like 2Fe-2S protein
*10 (groB)*	phenylpropionate dioxygenase-like ring-hydroxylating dioxygenase large terminal subunit
*11 (groC)*	Rieske-like 2Fe-2S protein
*12 (groD)*	farnesyl-diphosphate synthase
*13 (groH)*	hypothetical protein (prenyl transferase, guanidinatransferase)
*14 (groF)*	hypothetical protein (cyclase)
*15 (groG)*	protein-tyrosine-phosphatase

The functions of structural genes in *A. xinjiangensis* groBGC could be assigned based on the proposed gromomycin assembly pathway in *Streptomyces* sp. Je 1–332.^30^ *GroD* is coding for polyprenyl synthetase family protein. It performs the condensation of six isoprenoid precursors, forming a hexaprenyl pyrophosphate. Gene 13, homologue of *groH*, encodes a prenyltransferase, which is responsible for the hexaprenylguanidine formation. Gene 14, as a homologue of *groF*, encodes a hypothetical protein that serves as a gromomycin cyclase. While the genes 9, 10, and 11 are annotated as Rieske nonheme iron oxygenases, as their *groA*, *groB*, and *groC* homologues, they are also involved in the cyclization process by the introduction of the C4-OH group and the formation of C1–C2 and C15–C16 double bonds.^30^

The *gro*BGC of *A. xinjiangensis* lacks two genes, *groI* and *groE*, involved in tailoring modifications of gromomycin. The GroI cytochrome P450 monooxygenase introduces the keto group at the C17 position of the gromomycin C.^30^ While the GroE reductase catalyzes the reduction to a hydroxyl group, which, in turn, degrades, generating gromomycin A. Thus, we anticipate that the final products of the *A. xinjiangensis* groBGC are gromomycin I (480A1) and C (480A2). These derivatives are structurally similar, with the difference in the double bond position at C14–C15 for gromomycin I and at C15–C16 for gromomycin C (Figure 2B). We propose that both derivatives could arise during gromomycin cyclization, as terpene synthases (or cyclases) are known to facilitate carbocation-driven rearrangements, including intramolecular allylic rearrangements and double bond migration as part of their catalytic mechanisms.^34,35^ A similar mechanism is described during the cyclization reaction of a tetracyclic triterpene, euphol. An euphol-producing OSC enzyme (EtOSC5) is reported to produce two euphanes and two tirucallane triterpenoids. Both euphane (euphol and eupha-7,24-dien-3β-ol, 20R epimers) and tirucallane (tirucallol and tirucalla-7,24-dien-3β-ol, 20S epimers) derivatives are structural isomers distinguished by the double bond position at C7–C8 or C8–C9.^36^

### 3 Bioactivity Testing

We have previously reported that gromomycins display broad-spectrum activity against Gram-positive bacteria, including drug-resistant strains.^30^ Bioactivity testing of *A. xinjiangensis*-derived gromomycin derivatives revealed comparable antibacterial potencies, with gromomycins I and C being more active against the Gram-positive pathogens (2–4 μg/mL) than gromomycin J (16–32 μg/mL) (Table S5). Interestingly, while the position of the double bond in gromomycin C and gromomycin I does not appear to significantly influence antibacterial activity, its complete absence, as exemplified by gromomycin J, results in reduced activity. It is worth mentioning that, similar to previously described gromomycin derivatives, we observed a discrepancy between cell-based toxicity assays and the zebrafish embryo model. The zebrafish embryo model proved more sensitive toward gromomycin activity, with maximum tolerated concentrations (MTCs) closely mirroring antibacterial activity (Table S6). This discrepancy is likely due to binding to fetal bovine serum proteins present in the cell culture medium, which partly masks gromomycin toxicity (Table S7).

The promising activity of gromomycins prompted us to investigate their antibacterial potential further. Specifically, we tested one representative of this compound class, gromomycin I, against 89 vancomycin-resistant *E. faecium* (VRE) isolates, which also carry resistance determinants to multiple other antibiotics, underscoring their clinical relevance. Gromomycin I exhibited a unimodal distribution of MIC values, with MIC_50_ and MIC_90_ values of 2 μg/mL (Table 2), respectively, highlighting its ability to overcome resistance to conventional antibiotics.

**Table 2:** MIC_50_ and MIC_90_ of Gromomycin I and Reference Antibiotics among 89 Vancomycin-Resistant *E. faecium* Clinical Isolates^a^

		**MIC [μg/mL]**		
**antibiotic**	**EUCAST CBP**	**range** (*n* = 89)	**MIC**_**50**_	**MIC**_**90**_
gromomycin I	n/a	1 to 4	2	2
daptomycin	n/a	0.25 to 16	4	8
vancomycin	4	2 to >64	>64	>64
teicoplanin	2	0.5 to >64	64	>64
ramoplanin	n/a	0.25 to 4	2	2
linezolid	4	1 to 16	4	4
tigecycline	0.25	<0.03125 to 8	0.03125	0.25
ciprofloxacin	4	2 to >64	>64	>64
gentamicin	n/a	4 to >64	>64	>64
ampicillin	8	0.25 to >64	>64	>64

a CBP: clinical breakpoint. EUCAST: European Committee on Antimicrobial Susceptibility Testing. n/a: not available.

In addition, we assessed the killing kinetics of gromomycin I against *S. aureus* ATCC29213, which served as a model strain for subsequent mode of action studies. Gromomycin I demonstrated time- and concentration-dependent bactericidal activity. While 1× MIC led to a 2-log_10_ reduction in colony-forming units (CFUs)—likely attributable to the higher starting inoculum compared to that used in standard MIC assays, supra-MIC resulted in *a* >5-log_10_ reduction within only 30 min, indicating a potent and rapid bactericidal effect. Notably, the recovery of cells was observed across all tested concentrations approximately 7 h post-treatment (Figure 4A). However, this resurgence was not attributable to the development of spontaneous resistance, as isolated colonies remained susceptible to gromomycins. Indeed, gromomycins appear to have a low risk of resistance development. Attempts to select for gromomycin resistance in *S. aureus* ATCC29213 by plating high bacterial inocula on agar containing different doses of gromomycin I were unsuccessful. In addition, 30 days of continuous serial passaging in the presence of sub-MIC levels of gromomycin also failed to produce resistance. Similar results were obtained with the glycopeptide vancomycin, which targets the d-Ala-d-Ala motif of the bacterial peptidoglycan. In contrast, antibiotics with well-defined protein targets, such as rifampicin and ciprofloxacin, rapidly induced resistance within a few days of exposure (Figure 4B). The inability to select for gromomycin-resistant mutants strongly suggests a nonspecific mode of action and/or the absence of a conventional protein target.^37^

**Figure 4:**
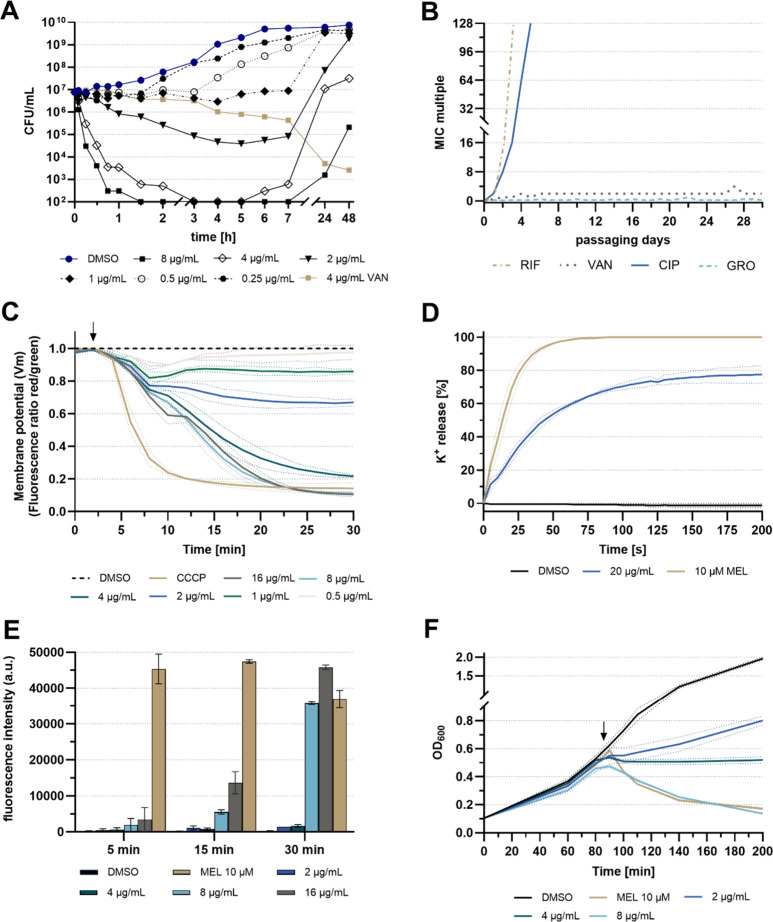
Gromomycin causes depolarization of the bacterial membrane and rapid cell lysis with bactericidal consequences for *Staphylococcus aureus*. (A) Time- and concentration-dependent killing of *S. aureus* by gromomycin I. The limit of detection is 10^2^ CFU/mL. Vancomycin was used as a positive control. (B) Resistance development occurs through serial passaging in the presence of sub-MIC levels of antibiotics. The *y-*axis represents the highest concentration with a visible growth. (C) Membrane depolarization assay was performed using DiOC_2_(3). The protonophore CCCP (5 μM) served as a positive control. Mean ± SD of two biological replicates (*n* = 2). The black arrow indicates the time point of compound addition. (D) Potassium released from whole cells. Potassium concentrations were measured using an ion-selective electrode. Leakage is expressed relative to the total amount of potassium release induced by the addition of 10 μM melittin. Mean ± SD of two biological replicates (*n* = 2). (E) Propidium iodide influx is an indicator of the presence of large membrane pores. The bee venom melittin is a membrane-disrupting peptide and was used as a positive control. Mean ± SD of two biological replicates (*n* = 2). (F) Impact of gromomycin I on the growth of the *S. aureus*. A decrease in the OD_600_ represents lysis of bacterial cells. The black arrow indicates the time point of compound addition. Mean ± SD of two technical replicates. A representative curve of two biological replicates is shown (*n* = 2). CIP, ciprofloxacin; GRO, gromomycin I; MEL, melittin; RIF, rifampicin; and VAN, vancomycin.

### 4 Mode of Action Studies

These findings, combined with the nonselective activity of gromomycins (Table S6) and their amphiphilic nature, point toward an interaction with the bacterial cell envelope. To test this hypothesis, we utilized the fluorescent dye 3,3′-diethyloxacarbocyanide iodide (DiOC_2_(3)), which serves as an indicator of membrane potential.^38^ In healthy polarized cells, DiOC_2_(3) emits red fluorescence, which shifts toward green fluorescence upon membrane depolarization. The addition of gromomycin I to DiOC_2_(3)-stained *S. aureus* cells induced bacterial membrane depolarization, as evidenced by a reduction in the red/green fluorescence ratio. Notably, this effect was observed at sub-MIC concentrations as low as 0.5–1 μg/mL (0.25–0.5× MIC) and increased gradually with rising concentration, ultimately reaching depolarization levels comparable to those induced by the protonophore carbonyl cyanide *m*-chlorophenyl hydrazone (CCCP) (5 μM). Importantly, the MIC-dependent depolarization of the molecule closely mirrors the bactericidal activity observed in the killing kinetics. A significant drop in membrane potential was observed between 2 μg/mL (1× MIC) and 4 μg/mL (2× MIC), corresponding to the onset of its bactericidal action (Figure 4A,C).

To investigate whether gromomycins enhance the ion permeability of the bacterial membrane, we measured the extracellular potassium concentration [K^+^] following gromomycin exposure using a K^+^-selective electrode. Addition of 20 μg/mL gromomycin I resulted in a rapid increase in extracellular [K^+^], reaching a maximum of 80% K^+^ release compared to the bee venom melittin, within approximately 2 min (Figure 4D). It is worth mentioning that the usage of high compound concentrations in this assay was necessary due to the high cell density (OD_600_ of 3) required to achieve detectable [K^+^]. The rapid onset of leakage, correlating with the fast depolarization and killing kinetics, prompted us to check for large pore formation. Indeed, we were able to confirm pore-forming activity by performing a propidium iodide influx assay. Propidium iodide is a fluorescent dye that enters the cells through large membrane pores and is thus indicative of membrane disruption. A strong increase in fluorescence was observed at concentrations starting at 8 μg/mL (4× MIC). In contrast to melittin, which promotes fluorescence immediately after 5 min, maximum values for gromomycin I were only observed after 30 min (Figure 4E). Consistent with these findings, we found that gromomycin I induces lysis of *S. aureus* cells at concentrations of 8 μg/mL (Figure 4F). However, pore formation and subsequent lysis occurred only at higher concentrations and were too slow to account for the much faster membrane depolarization, suggesting that these are secondary effects of gromomycin action.

Utilizing scanning and transmission electron microscopy (SEM and TEM), we investigated the impact of gromomycins on the morphology and intracellular structures of *S. aureus*. In control SEM samples, *S. aureus* cells appeared healthy with no visible damage. In contrast, after 15 min of exposure to supra-MIC doses of gromomycin I, cells started exhibiting signs of intracellular leakage, suggesting compromised membrane integrity (Figure S17). This effect became more apparent after 30 min, with almost all cells showing some form of membrane damage. *S. aureus* cells show membrane blebs, furrows, and dents, and even large pores are visible from which intracellular material is leaking (Figure 5A). TEM analysis provided further insights, revealing the presence of double-layered mesosomes and other aberrant membranous structures, along with numerous spherical, nonmembrane-enclosed vesicles in gromomycin-treated cells, which were not observed in control samples (Figure 5B). Mesosome-like structures arise from invaginations of the cytoplasmic membrane and can occur during fundamental bacterial processes, such as cell division. Although it is known that chemical fixation is a relevant factor for mesosomes, their formation has also been reported in response to antibiotic exposure.^38,39^ For example, lateral expansion of the lipid bilayer, driven by molecule insertion and the subsequent displacement of lipids, may result in the emergence of mesosome-like structures. Similar effects have been observed with the antimicrobial peptide gramicidin S, which is known to disrupt the lipid bilayer through interaction with membrane lipids. Interestingly, gramicidin S also promotes the emergence of inclusion bodies, likely composed of peptidoglycan precursors.^40^ Another explanation could be that these inclusion bodies are composed of lipids, potentially serving to compensate for the increased surface area of the bacterial cell membrane.

**Figure 5:**
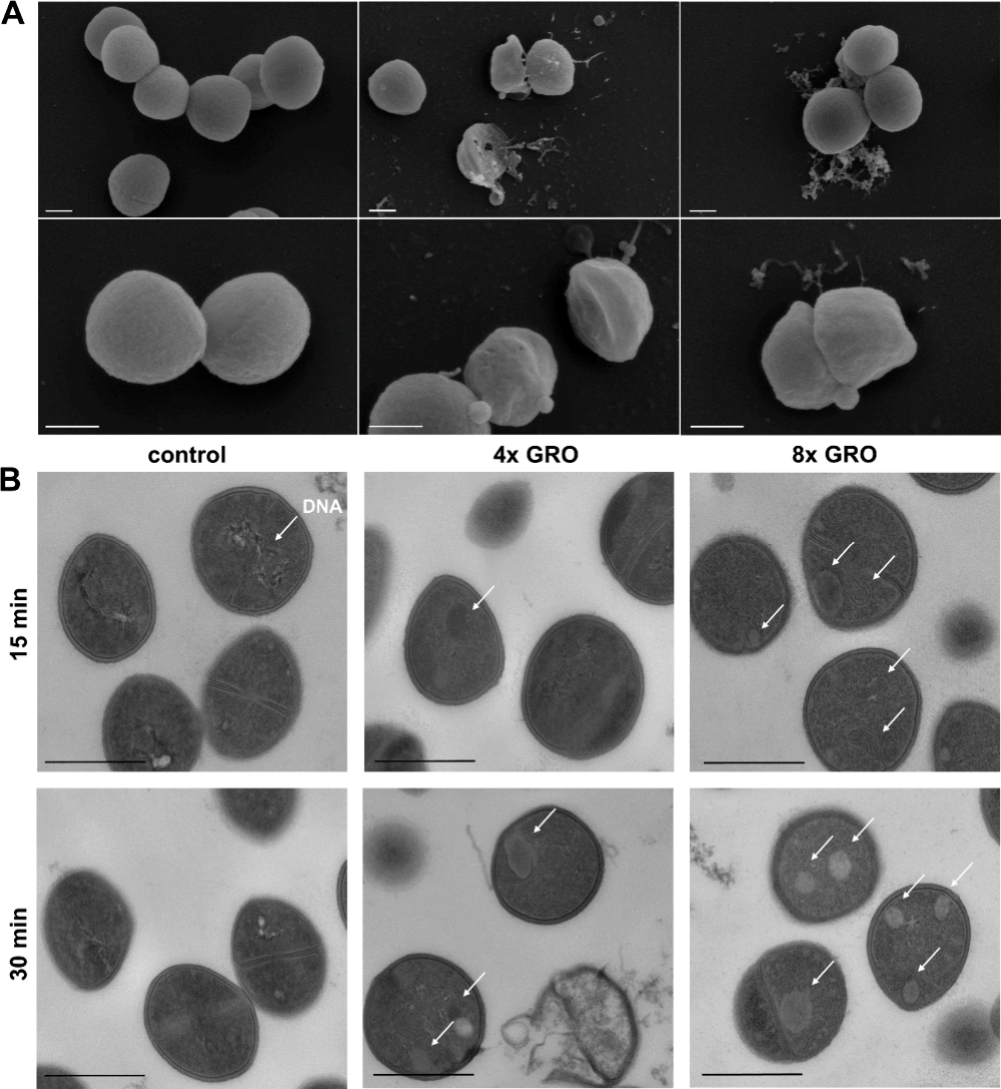
Ultrastructural analysis of *Staphylococcus aureus* exposed to gromomycin I. (A) Scanning electron microscopy (SEM) of *S. aureus* treated with 4- and 8-fold MIC of gromomycin I (*T* = 30 min). Scale bars are 300 nm. (B) Transmission electron microscopy (TEM) of *S. aureus* treated with 4- and 8-fold MIC of gromomycin I for 15 and 30 min, respectively. Arrows indicate irregularities induced by the compound that are not present in control samples. Scale bars are 500 nm. The control represents *S. aureus* cells exposed to DMSO. GRO, gromomycin I.

We next sought to investigate whether the compound’s effect on the bacterial cell envelope stems from a direct interaction with membrane lipids. To this end, we conducted MIC assays in the presence of representatives of fatty acids and lipids, monitoring for activity neutralization as a surrogate for lipid binding. MIC values shifted by up to 32-fold in a concentration-dependent manner, with the most significant changes induced by unsaturated fatty acids, such as palmitoleic acid, and negatively charged phosphatidylglycerols (PGs), including POPG (1-palmitoyl-2-oleoyl-*sn-glycero*-3-phosphatidylglycerol) and DOPG (1,2-dioleoyl-*sn-glycero*-3-phosphatidylglycerol). Neutral lipids resulted in a less pronounced shift (4- to 8-fold), and the cationic lipid DOTAP (1,2-dioleoyl-3-trimethylammonium propane) did not alter MIC values at all, likely due to electrostatic repulsion between this lipid and the positively charged gromomycin (Figure S18). To rule out the possibility that the observed effect is merely due to gromomycin’s positive charge, we included gentamicin, a positively charged antibiotic with an intracellular target, as a control. Notably, gentamicin’s activity remained unaffected in the presence of PGs and even increased upon the addition of fatty acids, suggesting a potential synergistic effect (Figure S18B).

Taken together, these results suggest that the antimicrobial activity of gromomycins primarily arises from membrane depolarization, driven by nonspecific interactions with membrane lipids and subsequent disruption of ion homeostasis. We postulate that gromomycins integrate into the membrane, a process facilitated by their amphiphilic scaffold: the cyclic hydrophobic part of the molecule likely interacts with the acyl chains of membrane lipids, while the positively charged guanidino group associates with the polar head groups. Electrostatic interactions between the guanidino moiety and anionic components of the cell envelope of Gram-positive bacteria, such as teichoic acids, likely contribute to the initial attraction to the bacterial surface.^41^ While it remains unclear whether gromomycins form an actual ion channel, we speculate that upon reaching a critical concentration, additional molecules insert into the membrane or oligomerize to form transient pores. This leads to the disruption of membrane integrity and ultimately results in cell lysis.

## Conclusions

The genome-mining approach has demonstrated impressive efficiency in the identification of novel gromomycin-like clusters, thereby highlighting their diversity among various bacterial taxa. This approach has resulted in the identification of a new *gro*BGC from the *Actinoplanes* species and the isolation of new bioactive gromomycin derivatives, highlighting the significant untapped potential of underexplored Actinomycetota in the field of antibiotic discovery. Mode of action studies have demonstrated that gromomycins act on the bacterial cell envelope. Targeting bacterial membranes has proven to be a highly effective antimicrobial strategy; however, it also raises concerns regarding toxicity toward eukaryotic cells. Due to the largely nonspecific nature of their interactions with lipid bilayers, membrane-active compounds can also disrupt eukaryotic cell membranes.^42–44^ Indeed, also in the case of gromomycins, the cytotoxicity closely mirrors their antibacterial activity. This off-target toxicity poses a major challenge in the clinical development of membrane-disrupting agents, calling for careful optimization of their selectivity index.^45^ Nevertheless, FDA-approved drugs such as gramicidin and colistin illustrate the clinical potential of this potent mechanism of action. Although gramicidin is limited to topical use due to its hemolytic activity,^46^ and colistin is reserved as a last-resort treatment for multidrug-resistant (MDR) infections due to its nephro and neurotoxicity,^47^ both compounds demonstrate that membrane-targeting agents can be successfully translated into clinical practice under carefully controlled conditions.

## Materials and Methods

### Bacterial Strains and Growth Media

All strains, plasmids, and primers used in this work are listed in Tables S2–S4. *A. xinjiangensis* DSM 45184 strain (Leibniz Institute DSMZ-German Collection of Microorganisms and Cell Cultures) was used as a source of DNA for cosmid library construction. *S. albus* Del14 and *S. lividans* ΔYA9 were utilized as hosts for heterologous expression of the new *gro*BGC. *Escherichia coli* EPI300-T1R cells were used for DSM 45184 cosmid library preparation. *E. coli* ET12567 pUB307 was used as a donor strain for intergeneric conjugations. *E. coli* strains were grown in Luria–Bertani (LB) broth (Sigma-Aldrich, St. Louis, MO, USA). *Streptomyces* strains were grown on MS agar medium (Soy flour 20 g, Mannitol 20 g, tap water 1 L, pH 7.2) and in liquid tryptic soy broth medium (TSB; Sigma-Aldrich, St. Louis, MO, USA). For genomic DNA isolation, the *A. xinjiangensis* strain was cultivated in liquid TSB medium. For conjugation, the *Streptomyces* strains were cultivated on MS agar for sporulation. Where necessary, the following antibiotics were applied: apramycin (50 μg/mL), kanamycin (50 μg/mL), and nalidixic acid (50 μg/mL) (Sigma-Aldrich, St. Louis, MO, USA; Roth, Karlsruhe, Germany). For compound production, S*treptomyces* strains were grown in liquid SG medium (20 g glucose, 10 g soy peptone, and 2 g CaCO_3_, distilled water 1 L, pH 7.2).

### Metabolite Extraction and Analysis

*S. albus* or *S. lividans* recombinant strains, along with *A. xinjiangensis* control, were cultivated in 50 mL of TSB medium for a period of 48 h at 28 °C, yielding a preculture. The main cultures containing 100 mL of SG, DNPM (40 g dextrin, 7.5 g soy peptone, 5 g baking yeast, and 21 g MOPS, distilled water 1 L, pH 6.8), or GYM (4 g glucose, 4 g yeast extract, 10 g malt extract, and 2 g CaCO_3_, distilled water 1 L, pH 7.2) were inoculated with 1 mL of preculture. Following a six-day cultivation period at 28 °C, the extraction of compounds was conducted using ethyl acetate from the clarified medium, followed by solvent evaporation. One μL of sample was measured using a Dionex Ultimate 3000 UPLC (Thermo Fisher Scientific, Waltham, MA, USA), a 10 cm ACQUITY UPLC BEH C18 column, 1.7 μm (Waters, Milford, MA, USA), and a linear gradient of 5%–95% of 0.1% formic acid (FA) solution in acetonitrile versus 0.1% FA solution in water for 18 min at a flow rate of 0.6 mL min^–1^ and 45 °C. Samples were analyzed using an amaZon Speed mass spectrometer or maXis high-resolution LC-QTOF system (Bruker, USA). Data were collected and analyzed with the Bruker Compass Data Analysis software, version 5.2 (Bruker, Billerica, MA, USA).

### Isolation and Purification of New Gromomycins

For production, the recombinant strain was grown in 10 L of SG medium for 6 days at 28 °C with agitation at 180 rpm. New derivatives were extracted with ethyl acetate from the culture supernatant. The obtained extracts were dissolved in methanol and subsequently subjected to a purification process via size-exclusion chromatography on a Sephadex LH-20 column (Sigma-Aldrich, St. Louis, MO, USA) with methanol as a mobile phase. Fractions were collected every 10 min at a flow rate of 0.6 mL min^–1^. The fractions containing pure compounds were pooled together, concentrated, and dissolved in methanol. The second purification stage was performed using a reversed phase (RP) HPLC (Waters AutoPurufication^TM^ System), separation on preparative C18 column Nucleodur HTec, 5 μm, 250 mm × 21 mm (Macherey-Nagel, Germany) using a water solution containing 0.1%(v/v) formic acid (solvent A), and an acetonitrile solution containing 0.1% (v/v) formic acid (solvent B) as a mobile phase. The fractions were collected using an MS detector (WatersTM SQ Detector 2). For compound separation, we used the following gradient at a flow rate of 20 mL/min: 0 min, 5% B; 1 min, 5% B; 2 min, 5% B; 4 min, 50% B; 25 min, 50.5% B; 27 min, 66% B; 29 min, 69% B; 30 min, 95% B; 31 min, 5% B. Gromomycin-containing fractions were pooled together, evaporated, and used for the final purification step. The final purification stage was reversed-phase High-performance liquid chromatography (HPLC), separation on a semipreparative C18 column SynergyTM 4 μm Fusion-RP 80 Å 250 × 10 (Phenomenex, Torrance, CA, USA) using water + 0.1% formic acid (A) and acetonitrile + 0.1% formic acid (B) as a mobile phase. Fractions containing the pure compound were pooled together and evaporated.

Gromomycin C white powder [11.4 mg], [α]_D_^20^ – 15° (c 0.53, MeOH). For NMR, see Table S1 (500 MHz, CD_3_OD). HRESIMS *m*/*z* 480.39 [M + H]^+^, (calcd for C_31_H_50_N_3_O, 480.3948).

Gromomycin I white powder [34.7 mg], [α]_D_^20^ – 41° (c 0.64, MeOH). For NMR, see Table S1 (500 MHz, CD_3_OD). HRESIMS *m*/*z* 480.39 [M + H]^+^, (calcd for C_31_H_50_N_3_O, 480.3948).

Gromomycin J white powder [9.6 mg], [α]_D_^20^ n.a. For NMR, see Table S1 (500 MHz, CD_3_OD). HRESIMS *m*/*z* 482.41 [M + H]^+^, (calcd for C_31_H_52_N_3_O, 482.4105).

### NMR Spectroscopy and Optical Rotation Measurements

The chemical structures of gromomycins were determined via multidimensional NMR analysis. ^1^H NMR, ^13^C NMR, and 2D spectra were recorded at 500 MHz (^1^H) and 126 MHz (^13^C), conducted in the Bruker Avance Neo 500 MHz, equipped with a Prodigy Cryo-probe. Gromomycins were dissolved in deuterated methanol*-d*_*4*_. All 2D experiments were measured using standard experiments from Bruker TopSpin software 4.3.0 and nonuniform sampling (NUS). Chemical shifts are reported in parts per million relative to tetramethylsilane; the solvent was used as the internal standard. Chiroptical measurements of all compounds in H_2_O ([α]_D_^20^) were obtained on a model Jasco P-2000 Automatic Digital Polarimeter (JASCO, Easton, MD, USA) in a 3.5 × 50 mm cell at 20 °C.

### Cosmid Library Construction

A cosmid library of actinomycetes was prepared using the EpiCentre CopyControl Fosmid Library Production Kit in the pCos15AAmInt vector by adapting the protocol from Lucigen. A library of 30–40 kb was constructed according to the protocol established by the manufacturer. Genomic DNA was isolated using the NucleoSpin Microbial DNA Mini kit (MACHEREY-NAGEL GmbH & Co. KG, Germany) for DNA isolation from microorganisms. Purified genomic DNA fragments were then ligated into the linearized cos15AAmInt vector. The ligation reactions were packaged into λ phages for *E. coli* EPI300 infection. The packaged library was plated on LB agar plates containing 50 μg mL^–1^ of apramycin and grown overnight at 37 °C. Approximately 1800 single colonies were picked and inoculated into individual wells of 96-well plates. The library was stored with 20% glycerol and kept at – 80 °C.

### Genome-Guided Identification of New Gromomycin-Like Clusters

A genome-wide quantitative screening of new gromomycins was conducted using three genes involved in the biosynthesis of gromomycins: *groD*, *groF*, and *groH*. The nucleotide sequences of these genes were used as probes in the NCBI protein BLAST database to identify new *gro*BGCs. The strains in which all three of these genes were found in close proximity in the genome were selected. As a result, many actinomycete strains were identified, including the *A. xinjiangensis* strain. The new *gro*BGC was isolated from the genome of the *A. xinjiangensis* strain through cosmid library construction according to the manual (CopyControl Fosmid Library Production Kit). The cosmid P19–C01 sample, which contained the entire cluster, was identified through a combination of end sequencing and PCR with two pairs of primers designed to amplify regions on the left and right sides of the cluster.

### Heterologous Expression of the New *gro*BGC

The cosmid 19-C01 harboring a novel *gro*BGC was introduced into *S. albus* Del14 and *S. lividans* ΔYA9 by a standard intergeneric mating protocol^48^ using donor strain *E. coli* ET12657 pUB307 on MS plates. After incubating at 29 °C for 15 h, plates were overlaid with 1 mL of sterile distilled water containing 50 μg mL^–1^ apramycin and 50 μg mL^–1^ nalidixic acid. Antibiotic-resistant transconjugants were patched onto MS plates containing 50 μg mL^–1^ apramycin. The ability of heterologous strains to generate novel derivatives was assessed through HPLC analysis.

### Antibiotic Activity (Minimum Inhibitory Concentrations)

Gromomycin stock solutions were prepared in dimethyl sulfoxide (DMSO). All microorganisms used in this study were obtained from the German Collection of Microorganisms and Cell Cultures (DSMZ), the American Type Culture Collection (ATCC), and the Coli Genetic Stock Center, or were part of our internal strain collection. *S. aureus* strains Newman, N315, Mu50, and Cowan 1 were obtained from M. Bischoff, Saarland University Hospital, Homburg. *S. aureus* wild type and Dap^R^ HG001 were provided by T. Schneider, University of Bonn.^49^ *E. coli* WO153 was provided by K. Lewis, Northeastern University, Boston, USA. *E. faecium* clinical isolates were collected between 2019 and 2024 and were provided by Stefano Mancini, Institute of Medical Microbiology, Zürich, Switzerland. Minimum inhibitory concentrations (MICs) were determined using the broth microdilution method according to EUCAST guidelines (ISO 20776-1:2019). In short, serial 2-fold dilutions of gromomycins (0.03125 to 64 μg/mL) were prepared in 75 μL of cation-adjusted Mueller-Hinton broth (MHB2) in sterile 96-well plates. An equal volume of the bacterial suspension was added, and the plates were incubated at 37 °C for 18 h. For *Streptococcus pneumoniae*, MHF broth (MHB2 supplemented with 5% lysed horse blood and 20 mg/L β-NAD) was used, and plates were incubated at 37 °C with 5% CO_2_. The MIC was defined as the lowest concentration of the antibiotic causing complete inhibition of visible growth of the microorganism. The same method was used for testing *Mycobacterium smegmatis*, but with the use of Middlebrook 7H9 complete medium supplemented with oleic acid, albumin, dextrose, and catalase (OADC, 10%). *M. smegmatis* plates were incubated for 48 h at 37 °C. For assessing activity against *M. tuberculosis*, an adapted resazurin microtiter assay (REMA) was performed. In short, *M. tuberculosis* single cells were prepared and added to compound dilutions in M7H9. Plates were incubated for 6 days at 37 °C, followed by the addition of 50 μL of resazurin and incubation for another day at 37 °C. The MIC was determined visually and additionally confirmed by measuring fluorescence (excitation at 530 nm, emission at 590 nm).

### Cytotoxic Activity (IC50)

HepG2 cells (human hepatoblastoma cell line; ACC 180) and CHO-K1 cells (Chinese hamster ovary cells; ACC 110) were obtained from the German Collection of Microorganisms and Cell Cultures (DSMZ) and cultured under the conditions recommended by the depositor. Cells were propagated in Roswell Park Memorial Institute (RPMI) 1640 medium and Ham’s F12 medium, respectively, supplemented with 10% fetal bovine serum (FBS), and seeded at 6 × 10^3^ cells per well of 96-well plates in 120 μL of complete medium. After 2 h of equilibration (37 °C, 5% CO_2_), the cells were treated with a serial dilution of the gromomycins. Gromomycins, doxorubicin as a reference, and the solvent control (DMSO), were tested in duplicate in two independent experiments. After 5 d of incubation (37 °C, 5% CO_2_), a total of 20 μL of 5 mg mL^–1^ MTT (thiazolyl blue tetrazolium bromide) in phosphate-buffered saline (PBS) was added to each well, and the cells were further incubated for 2 h at 37 °C before the supernatant was discarded. Subsequently, the cells were washed with 100 μL of PBS and treated with 100 μL of 2-propanol/10 N HCl (250,1) to dissolve formazan granules. Cell viability was measured as a percentage relative to the respective solvent control by measuring the absorbance at 570 nm using a microplate reader (Tecan Infinite M200Pro). GraphPad Prism (version 10.0.3, GraphPad, Boston, MA, USA) was used for sigmoidal curve fitting to determine the IC_50_ values.

### Maximum Tolerated Concentration

Husbandry of adult zebrafish was performed according to internal guidelines set out in the German Animal Welfare Act (§11 Abs. One TierSchG). Experiments were carried out with wild-type AB (obtained from the European Zebrafish Resource Center at Karlsruhe Institute of Technology) embryos within the first 120 h post fertilization (hpf), as these early life stages are not considered animal experiments according to the EU Directive 2010/63/EU. Embryos were maintained in fresh 0.3× Danieau’s solution (17.4 mM NaCl, 0.21 mM KCl, 0.12 mM MgSO_4_, 0.18 mM Ca (NO_3_)_2_, 1.5 mM HEPES, 1.2 μM methylene blue, pH 7.1–7.3) at 28 °C. At a maximum of 120 hpf, embryos were euthanized by submersion in ice water for at least 12 h. For evaluation of the maximum tolerated concentration (MTC), embryos were dechorionated at 30 hpf using 1 mg mL^–1^ Pronase and placed in a flat-bottom 96-well plate with one embryo per well. Excess medium was removed, and 150 μL of gromomycin dilutions (in 0.3× Danieau’s, maximum of 1% DMSO) and of the solvent control (1% DMSO in 0.3× Danieau’s) were added. Ten embryos were used per condition. Exposed embryos were maintained at 28 °C until 120 hpf, and they were monitored daily under a stereo microscope (Stemi 508, Zeiss) in order to record survival as well as anomalies, pigmentation, heartbeat, and locomotor responses. An embryo was considered dead when no heartbeat could be observed. The maximum tolerated concentration (MTC) was defined as the highest concentration of the antibiotic with more than 90% survival of zebrafish embryos. Kaplan–Meier curves were generated using GraphPad Prism (version 10.0.3, GraphPad, Boston, MA, USA).

### Time-Kill Kinetics

An overnight culture of *S. aureus* ATCC29213 was diluted 1:100 in MHB2 and incubated at 37 °C until the log phase was reached. The bacteria were adjusted to reach approximately 10^7^ CFU/mL, distributed to test tubes, and challenged with different concentrations of gromomycin I. DMSO was used as a negative control, and 2× MIC of vancomycin was used as a positive control. The bacteria were incubated at 37 °C and 300 rpm, and at designated time points, an aliquot of the samples was taken, and appropriate dilutions were plated on CASO agar. Agar plates were incubated at 37 °C overnight, and colonies were counted to calculate CFU/mL.

### Resistance Studies

For single-step resistance, CASO agar plates containing 4× and 8× MIC of gromomycin I were prepared. 5 × 10^9^ and 5 × 10^8^ CFU (*S. aureus* ATCC29213) were plated on selective agar plates, and appropriate dilutions of the inoculum were plated on nonselective agar to determine the proper count. Plates were incubated for 48 h at 37 °C. For resistance development by serial passaging, an overnight culture of *S. aureus* ATCC29213 was diluted 1:200 in fresh MHB2 containing different compound concentrations (0.25× to 4× MIC). Test tubes were incubated at 37 °C overnight, and the next day, the highest concentration with visible growth was used to inoculate (1:200) the next series of concentrations based on the growth results from the previous day. Rifampicin, ciprofloxacin, and vancomycin were used as reference compounds. This procedure was repeated until a significant level of resistance was reached or terminated after 30 days. Resistance was confirmed by the broth microdilution method.

### Electron Microscopy

An overnight culture of *S. aureus* ATCC 29213 was subcultured 1:100 in fresh MHB2 and reincubated until OD_600_ 0.5 was reached. The culture was divided into 2 mL samples and exposed to 4× and 8× MIC of gromomycin I or DMSO (control). Samples were incubated for 15 and 30 min at 37 °C and 300 rpm, respectively. Cells were fixed by incubating with 2% glutaraldehyde and 5% paraformaldehyde (final concentrations) for 30 min. Samples for both SEM and TEM were processed as previously described.^50^ For TEM, samples were further treated with osmium tetroxide, dehydrated in a graded series of ethanol on ice, and embedded in LR White resin. 50–70 nm thick, ultrathin sections were counterstained with 4% aqueous uranyl acetate and lead citrate and analyzed with a Libra 120 Plus instrument (Zeiss, Oberkochen, Germany) operating at an acceleration voltage of 120 kV and with the image analysis software ITEM (Olympus). For SEM, bacteria were fixed to 12 mm, round, poly-l-lysine pretreated coverslips and dehydrated in a graded series of acetone on ice, critical-point dried with liquid CO_2_ (CPD 300, Leica Microsystems, Wetzlar) and sputter coated with a gold–palladium film (SCD 500, Bal-Tec, Lichtenstein). Image acquisition was performed with a Zeiss Merlin field-emission scanning electron microscope (Oberkochen, Germany) using Everhart Thornley and the In-lens SE detectors at an acceleration voltage of 5 kV.

### Membrane Potential Assay

The assay was performed as previously described. An overnight culture of *S. aureus* ATCC29213 was diluted 1:100 in fresh LB medium supplemented with 0.1% glucose and incubated at 37 °C until the log-phase was reached. Cells were pelleted (4000 rpm, 4 °C, 5 min) and resuspended in PBS supplemented with 0.1% glucose to an OD_600_ of 0.5. Cells were incubated with 30 μM 3,3′-diethyloxacarbocyanine iodide (DiOC_2_(3)) for 15 min in the dark. DiOC_2_(3)-treated cells were transferred to a black-bottom 96-well plate with 100 μL/well, and a baseline measurement was recorded for 4 min at an excitation wavelength of 485 nm and emission wavelengths of 530 (green) and 630 nm (red). The measurement was stopped and a concentration series of gromomycin I (1 μL of 100× stocks) was added, after which the measurement was continued for a total of 30 min. Carbonylcyanide-*m*-chlorphenylhydrazone (5 μM, CCCP) was used as a positive control, while 1% DMSO served as a negative control. Experiments were performed in technical and biological duplicates.

### Potassium Release from Whole Cells

Potassium release was measured using a potassium-selective electrode as recently described, with a few modifications.^51^ An overnight culture of *S. aureus* ATCC29213 was diluted 1:100 in fresh MHB2 and incubated at 37 °C until the early stationary phase (OD_600_ 1.2) was reached. Cells were pelleted (4000 rpm, 4 °C, 20 min) and washed with 10 mM Tris–HCl, 100 mM NaCl, pH 7.4, adjusted with ionic strength adjuster (2/100 mL buffer). The cell suspension was pelleted again, and the pellet was resuspended in the aforementioned buffer to reach an OD_600_ value of 30. The bacteria were kept on ice and used within 30 min. The potassium electrode was calibrated with standard solutions containing 10, 100, and 1000 mg/L K^+^, respectively. For each measurement, the bacteria were diluted 1:10 with buffer, and the baseline potassium concentration was measured ([K^+^]_init_). Then, 10× MIC of gromomycin I (20 μg/mL) or 10 μM melittin was added, and potassium release was measured every 5 s until the potassium value remained stable ([K^+^]_meas_). DMSO was used as a negative control. Experiments were performed in biological duplicates. Leakage is expressed relative to the total amount of potassium release induced by the addition of 10 μM melittin ([K^+^]_tot/MEL_).

$$K^{+}\;\mathrm{release}\;[\%]=\frac{[K^{+}{]}_{\mathrm{meas}}-[K^{+}{]}_{\mathrm{init}}}{[K^{+}{]}_{\mathrm{tot}/\mathrm{MEL}}-[K^{+}{]}_{\mathrm{init}/\mathrm{MEL}}}\times 100$$

### Propidium Iodide Influx Assay

An overnight culture of *S. aureus* ATCC29213 was subcultured 1:100 in fresh MHB2 and reincubated until OD_600_ 0.5 was reached. Bacteria were treated with 1×, 2×, 4×, or 8× MIC of gromomycin I, 10 μM melittin (positive control), or DMSO in test tubes in a total volume of 500 μL. Samples were incubated at 37 °C and 300 rpm, and after 5, 15, and 30 min, 100 μL aliquots were taken and stained with 10 μg/mL propidium iodide for 5 min in the dark (37 °C, 300 rpm). Stained bacteria were pelleted (maximum speed, 2 min, 4 °C) and washed twice with 100 μL of PBS. The pellet was resuspended in PBS, and cells were dispensed in a black flat-bottom 96-well plate. Fluorescence was measured at an excitation wavelength of 535 nm and an emission wavelength of 617 nm. Experiments were performed in technical triplicate and biological duplicates.

### Lysis Assay

An overnight culture of *S. aureus* ATCC29213 was subcultured 1:100 in fresh MHB2 and reincubated until OD_600_ of 0.5 was reached. Bacteria were diluted to an OD_600_ of 0.1 and distributed into test tubes, after which they were grown to mid-log phase. Subsequently, 1×, 2×, or 4× MIC of gromomycin I, 10 μM melittin (positive control), or DMSO (negative control) were added. OD_600_ was measured until 120 min after the compound addition. Experiments were performed in technical and biological duplicates.

## Supplementary Material



## Notes

Additional experimental details, materials, and methods; NMR spectra and structural elucidation data; antibacterial and cytotoxicity data; SEM and TEM micrographs; and figures illustrating lipid interaction and resistance assays (PDF)
