# Supplementary material for: Genome Mining-Driven Isolation of New Gromomycins and Insights into Their Mode of Action
Source: ACS Chem Biol. 2026 Mar 5;21(3):546–57. doi: 10.1021/acschembio.5c00821 (PMC13010261; doi:10.1021/acschembio.5c00821)
Supplement: Supplementary file 1 [file cb5c00821_si_001.pdf]

Supplementary Information

Genome Mining–Driven Isolation of New Gromomycins and Insights into Their Mode of Action

Dmytro Bratiichuk[1]*, Franziska Fries[2,3]*, Marc Stierhof[1], Leon Morguet[2], Josef Zapp[1,2], Mathias Müsken[4], Yuriy Rebets[5], Maksym Myronovskyi[1], Rolf Müller[2,3], Jennifer Herrmann[2,3#], Andriy Luzhetskyy[1,2#]

[1]Department of Pharmaceutical Biotechnology, Saarland University, Bld. C2.3, 66123 Saarbrücken, Germany.

[2]Helmholtz Institute for Pharmaceutical Research Saarland (HIPS), Helmholtz Centre for Infection Research (HZI), Saarland University, 66123 Saarbrücken, Germany.

[3]German Center for Infection Research (DZIF), Partner Site Hannover-Braunschweig, 38124 Braunschweig, Germany.

[4]Helmholtz Centre for Infection Research (HZI), 38124 Braunschweig, Germany.

[5]German-Ukrainian Core of Excellence in Natural Products Research (CENtR), Zelena st. 20, Lviv 79005, Ukraine

*Equally contributed to this work

# Corresponding authors: a.luzhetskyy@mx.uni-saarland.de; jennifer.herrmann@helmholtz-hips.de

Table of contents

Structure elucidation 3

List of Tables 3

Table S1: NMR data (500MHz, MeOD4) of gromomycin C, gromomycin I and gromomycin J. 3

Table S2. Bacterial strains used in this work. 5

Table S3. Plasmids used in this work. 5

Table S4. Primers used in this work. 5

Table S5. Antimicrobial activity spectrum of gromomycin derivatives. 6

Table S6. Toxicity profile of gromomycin derivatives. 7

Table S7. Gromomycin I binds to plasma proteins. 8

List of Figures 9

Figure S1: Structures of gromomycins C, I and J, showing COSY correlations and key HMBC correlations crucial for the assignment of ring D. 9

Figure S2: ^{1}H-NMR spectrum (500 MHz, $MeOD_4$) of gromomycin C (480A2). 10

Figure S3: ^{13}C-NMR spectrum (125 MHz, $MeOD_4$) of gromomycin C (480A2). 10

Figure S4: COSY spectrum ($MeOD_4$) of gromomycin C (480A2). 11

Figure S5: HSQC spectrum ($MeOD_4$) of gromomycin C (480A2). 12

Figure S6: HMBC spectrum ($MeOD_4$) of gromomycin C (480A2). 13

Figure S7: ^{1}H-NMR spectrum (500 MHz, $MeOD_4$) of gromomycin I (480A1). 14

Figure S8: ^{13}C-NMR spectrum (125 MHz, $MeOD_4$) of gromomycin I (480A1). 15

Figure S9: COSY spectrum (500 MHz, $MeOD_4$) of gromomycin I (480A1). 16

Figure S10: HSQC spectrum ($MeOD_4$) of gromomycin I (480A1). 17

Figure S11: HMBC spectrum ($MeOD_4$) of gromomycin I (480A1). 18

Figure S12: ^{1}H-NMR spectrum (500 MHz, $MeOD_4$) of gromomycin J (482A). 19

Figure S13: ^{13}C-NMR spectrum (125 MHz, $MeOD_4$) of gromomycin J (482A). 20

Figure S14: COSY spectrum ($MeOD_4$) of gromomycin J (482A). 21

Figure S15: HSQC spectrum ($MeOD_4$) of gromomycin J (482A). 22

Figure S16: HMBC spectrum ($MeOD_4$) of gromomycin J (482A). 23

Figure S17. Bacterial phenotyping by scanning electron microscopy (SEM) imaging. 24

Figure S18. Gromomycin activity is impaired in the presence of fatty acids and phospholipids. 25

Figure S19. Mass spectra analysis (m/z) of Gromomycins I, C, ... 25

Figure S20. All *gro*BGCs identified with CluSeek. Genes groD, groF and groH, representing the core part of gromomomycin BGC are highlighted in red. 26

References 27

Structure elucidation

List of Tables

Table S1: NMR data (500MHz, MeOD4) of gromomycin C, gromomycin I and gromomycin J.

	gromomycin C (480A2)*		gromomycin I (480A1)		gromomycin J (482A)	
No	δ(^{13}C) [ppm], type	δ(^{1}H) [ppm], mult(J)	δ(^{13}C) [ppm], type	δ(^{1}H) [ppm], mult(J)	δ(^{13}C) [ppm], type	δ(^{1}H) [ppm], mult(J)
1	116.4, CH	5.92, s	116.1, CH	5.89, s	116.3, CH	5.92, s
2	132.7, C	-	132.3, C	-	132.5, C	-
3	59.9, C	-	59.9, C	-	59.9, C	-
4	80.0, CH	3.68, dd (10.7, 4.0)	79.6, CH	3.70, dd (11.3, 4.5)	79.5, CH	3.68, dd (11.7, 4.5)
5	29.6, CH_2	1.60, m<br>1.86, m	29.2, CH_2	1.73, m<br>1.59, m	27.8, CH_2	1.82, m<br>1.52, m
6	47.4, CH	1.57, m	41.8, CH	1.64, m	56.9, CH	1.06, m
7	38.0, C	-	38.4, C	-	39.0, C	-
8	35.2, CH_2	1.51, m	35.8, CH_2	1.53, m<br>1.24, m	40.9, CH_2	1.87, m<br>1.37, m
9	34.2, CH_2	1.82, m<br>1.74, m	28.1, CH_2	1.70, m<br>1.58, m	41.0, CH_2	1.72, m<br>0.81, m
10	43.7, C	-	43.4, C	-	39.2, C	-
11	39.2, C	-	38.6, C	-	38.8, C	-
12	33.6, CH_2	1.39, m<br>1.66, m	31.3, CH_2	1.56, m<br>1.49, m	42.9, CH_2	1.83, m<br>0.95, m
13	20.5, CH_2	1.46, m<br>1.54, m	22.5, CH_2	1.93, m<br>1.69, m	18.5, CH_2	1.67, m<br>1.50, m
14	41.8, CH	2.35, m	135.4, C	-	54.7, CH	0.93, m
15	145.1, C	-	136.5, C	-	62.7, CH	0.82, m
16	122.6, CH	5.67, m	27.7, CH_2	2.09, m<br>1.82, m	19.6, CH_2	1.47, m<br>1.69, m
17	24.6, CH_2	2.1, m	21.3, CH_2	1.52, m<br>1.69, m	19.1, CH_2	1.48, m
18	34.6, CH_2	1.47, m<br>1.38, m	36.5, CH_2	1.61, m<br>1.27, m	38.5, CH_2	1.30, m
19	36.4, C	-	39.5, C	-	36.7, C	-
20	42.3, CH_2	1.28, m	41.8, CH_2	1.56, m<br>1.08, m	45.5, CH_2	1.25, m<br>1.10, m
21	23.4, CH_2	1.91, m	24.9, CH_2	2.02, m<br>1.56, m	23.1, CH_2	1.87, m
22	126.5, CH	5.09, m	126.5, CH	5.02, m	126.4, CH	5.08, m
23	131.8, C	-	132.0, C	-	131.8, C	-
24	17.9, CH_3	1.59, s	18.1, CH_3	1.54, s	26.1, CH_3	1.67, s
25	23.8, CH_3	1.40, s	24.0, CH_3	1.39, s	23.7, CH_3	1.39, s
26	25.3, CH_3	1.21, s	25.0, CH_3	1.20, s	26.1, CH_3	1.18, s
27	29.8, CH_3	1.03, s	24.1, CH_3	0.92, s	17.4, CH_3	0.91, s
28	22.5, CH_3	0.85, s	21.3, CH_3	0.86, s	18.4, CH_3	0.92, s

29	19.1, CH_3	0.76, s	28.9, CH_3	1.02, s	21.6, CH_3	0.83, s
30	26.1, CH_3	1.66, s	26.1, CH_3	1.66, s	17.8, CH_3	1.59, s
1'	152.9, C	-	152.6, C	-	152.8, C	-

*The literature data of gromomycin C were measured in DMSO-d_6. For better comparability, a small sample of gromomycin C (480A2) was also measured in DMSO-d_6. The ^{1}H and HSQC data obtained were consistent with the literature data.

Table S2. Bacterial strains used in this work.

Strains	Description	Reference or Source
Actinoplanes xinjiangensis DSM 45184	Type strain	DSMZ-German Collection of Microorganisms and Cell Cultures
S. albus Del14	Cluster-free heterologous host strain	[1]
S. lividans ΔYA9	Cluster-free heterologous host strain	[2]
S. albus Del14 cos15AAmInt-19C01	S. *albus* strain Del14 containing the cos15AAmInt-19C01 vector	This work
S. lividans ΔYA9 cos15AAmInt-19C01	*S. lividans* ΔYA9 containing the cos15AAmInt-19C01 vector	This work
E. coli ET12567 pUB307	Donor strain for intergeneric conjugation	[3]
E.coli EPI300-T1R	Strain used for Construction of inducible-copy-number genomic libraries using the CopyControl™ Cloning System, with clones that are resistant to contaminating phage T1 and T5	Lucigen

Table S3. Plasmids used in this work.

Plasmids	Description	Reference or Source
pCos15A_AmInt	pCos15A_gus_AmInt, where *gus* gene was deleted	[4]
Cos15AAmInt-19C01	Cosmid 19C01 containing gromomycin-like gene cluster with *aac(3) IV*, *oriT*, and integrase	This work

Table S4. Primers used in this work.

Primer name	Sequence (5'-3')	Description	Source
actino_7_ F<br>actino_7_R	CTGCGTCCTGTTCTGGATGA<br>CACGGGTTCTTTCCCTCGG	To test for *groF* homolog gene presence	This work
Left_actino_F<br>Left_actino_R	GAGACGATCAGCACCTCCG<br>GACAACGGCCGCTCCTAC	To sequence the insert containing gromomycin-like cluster from the left side of the cluster	This work
Right_actino_F<br>Right_actino_R	CTGGCAACTGCTCCATGAG<br>GGAACGGTGGATAGTCGTCG	To sequence the insert containing gromomycin-like cluster from the right side of the cluster	This work

Table S5. Antimicrobial activity spectrum of gromomycin derivatives.

Classification	Organism	MIC [µg/mL]			
		Gromomycin I	Gromomycin C	Gromomycin J	REF[c]
Gram-positive	*S. aureus* ATCC29213	2	4	32	VAN: 2
	S. aureus Newman	2	2	16-32	VAN: 2
	S. aureus Cowan 1	2	4	32	VAN: 2
	S. aureus N315	2	4	32	VAN: 2, AMP: > 64
	S. aureus Mu50	2	4	16	VAN: 8, AMP: > 64
	S. aureus HG001 WT	2	4	32	DAP: 1
	S. aureus HG001 Dap[R]	2	2	8	DAP: 64
	S. pneumoniae DSM11865	2	2	8	RIF: 0.03
	E. faecalis ATCC29212	2	4	32	RIF: 0.5
	B. subtilis DSM10	2	2	32-64	VAN: 1
Mycobacteria	*M. smegmatis* mc^{2}155	16	16	16	RIF: 32
	M. tuberculosis H37Ra	4	4	4-8	RIF: 0.02
Gram-negative	*E. coli* BW25113	> 64	> 64	> 64	CIP: 0.03
	E. coli K12 Δ*tolC*[a]	> 64	> 64	> 64	CIP: 0.01
	E. coli K12 Δ*tolC*[a] + PMBN[b]	4	4	64	CIP: 0.01
	E. coli WO153	4	32	32	CIP: 0.01
	S. enterica DSM5569	> 64	> 64	> 64	CIP: 0.02
	C. freundii DSM30039	> 64	> 64	> 64	CIP: 0.02
	K. pneumoniae DSM681	64	> 64	> 64	CIP: 0.01
	A. baumannii DSM30007	16	> 64	> 64	CIP: 2
	A. baumannii DSM30008	16	> 64	> 64	CIP: 0.5
	A. baumannii NCTC13301	16-32	> 64	> 64	CIP: > 64, COL: 1
	P. aeruginosa PA14	> 64	> 64	> 64	CIP: 0.25

[a] Keio collection mutant; efflux-deficient [b] 3 µg mL^{-1} polymyxin B nonapeptide (PMBN) [c] Reference antibiotics: AMP, ampicillin; CIP, ciprofloxacin; COL, colistin; DAP, daptomycin; RIF, rifampicin; VAN, vancomycin

[R] Daptomycin resistance

Table S6. Toxicity profile of gromomycin derivatives. *In vitro* cytotoxicity was evaluated performing a MTT assay (half maximal inhibitory concentration IC_{50} ± standard deviation). *In vivo* toxicity was assessed by determination of the maximum tolerated concentration (MTC) in zebrafish (*Danio rerio*) embryos. CHO: chinese hamster ovary.

**Gromomycin derivative**	***In vitro* IC_{50} [µg/mL]**		***In vivo* MTC [µg/mL]**
	**HepG2**	**CHO-K1**	***Danio rerio***
**Gromomycin I**	24.5 ± 8.3	25.4 ± 3.3	1
**Gromomycin C**	22.7 ± 0.2	24.3 ± 1.3	1
**Gromomycin J**	22.7 ± 2.0	> 37	nd[a]

[a] not determined (nd) due to poor solubility in the incubation medium

Table S7. Gromomycin I binds to plasma proteins. Minimum inhibitory concentrations (MICs) of gromomycin I and daptomycin in the presence of different plasma protein constituents. MICs were determined in MHB2 adjusted to 1.25 mM Ca^{2+} and 0.8 mM Mg^{2+} (physiological concentration). BSA: bovine serum albumin; FBS: fetal bovine serum

**Condition**	**MIC [µg/mL]**	
	**Gromomycin I**	**Daptomycin**
**MHB2 (no addition)**	2	1
**6% (*w/v*) BSA**	32	16
**25% (*v/v*) FBS**	8	1
**50% (*v/v*) FBS**	8	1
**25% (*v/v*) human serum**	32	2
**50% (*v/v*) human serum**	64	4

List of Figures

Figure S1: Structures of gromomycins C, I and J, showing COSY correlations and key HMBC correlations crucial for the assignment of ring D.

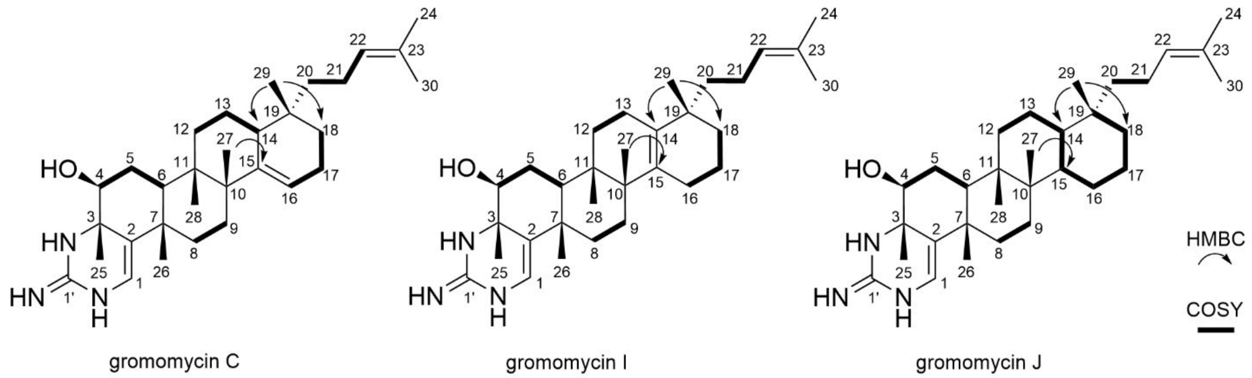

Figure S2: ^{1}H-NMR spectrum (500 MHz, $MeOD_4$) of gromomycin C (480A2).

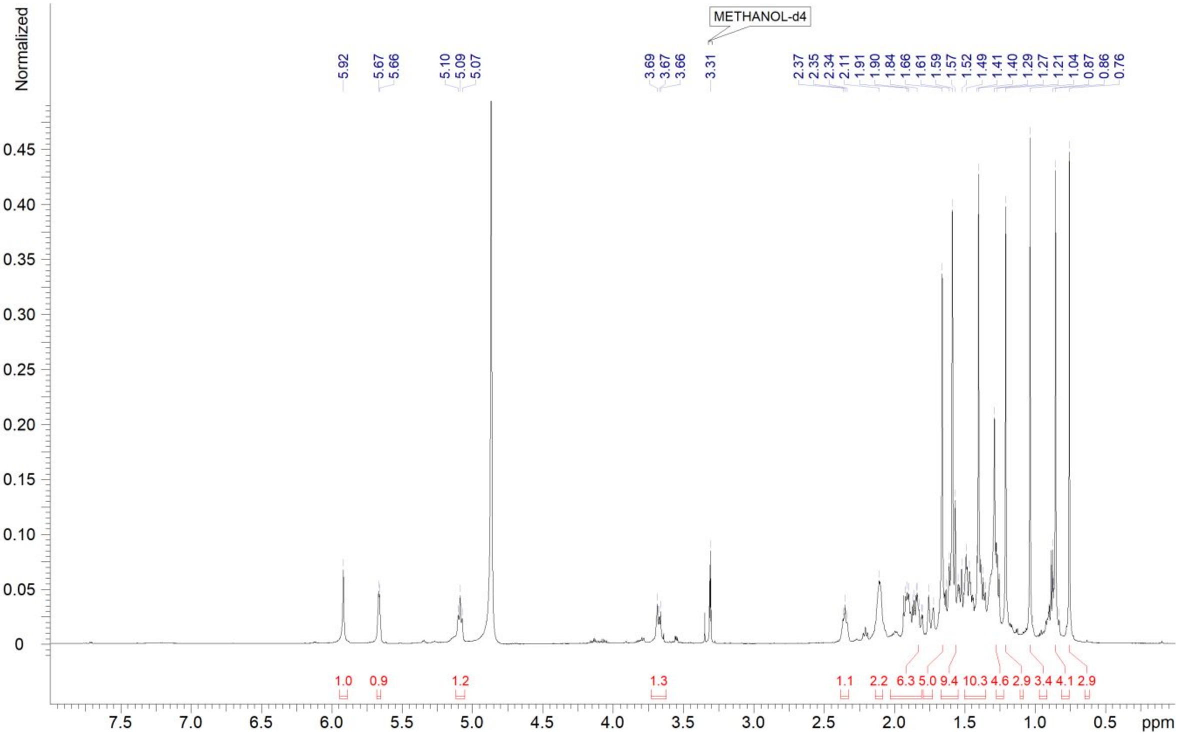

Figure S3: ^{13}C-NMR spectrum (125 MHz, $MeOD_4$) of gromomycin C (480A2).

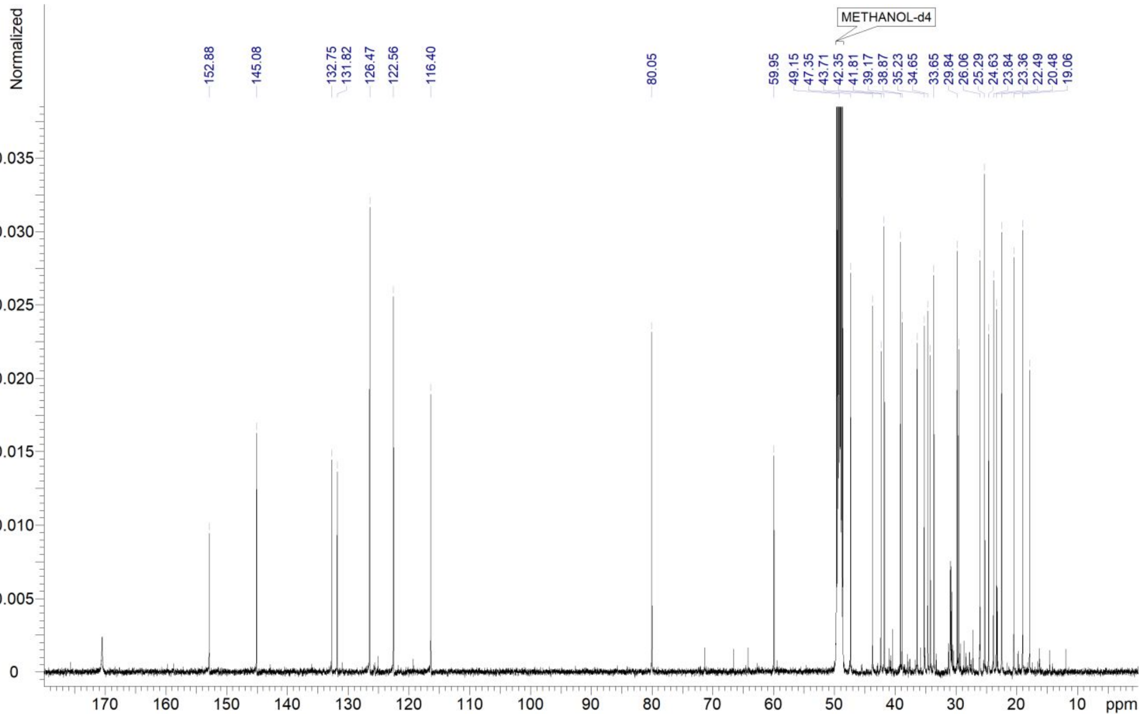

Figure S4: COSY spectrum (MeOD$_4$) of gromomycin C (480A2).

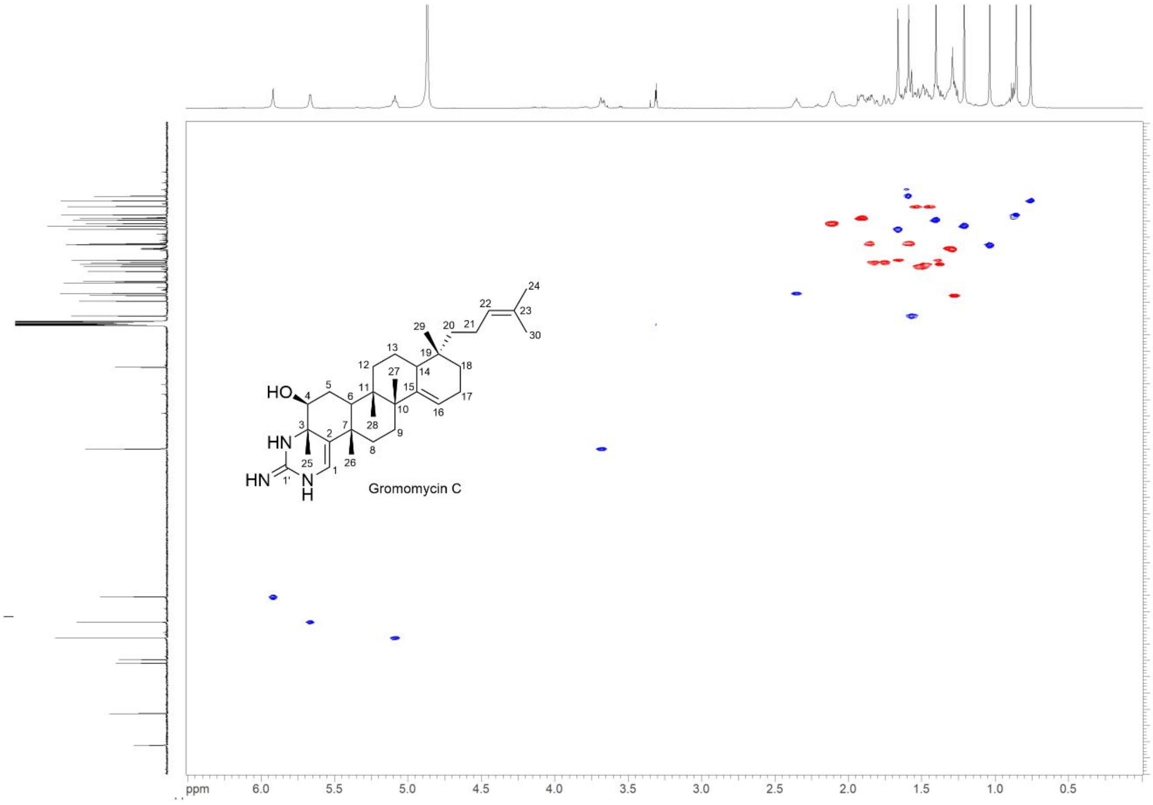

Figure S5: HSQC spectrum ($MeOD_4$) of gromomycin C (480A2).

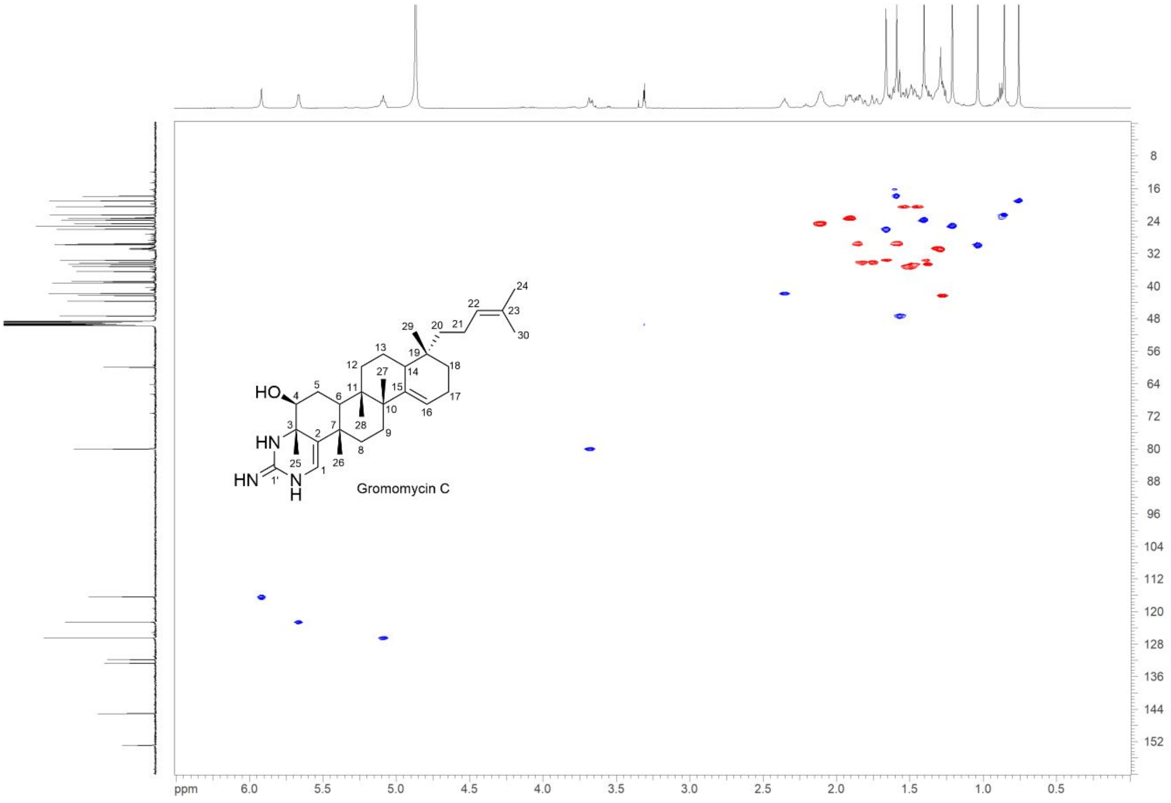

Figure S6: HMBC spectrum ($MeOD_4$) of gromomycin C (480A2).

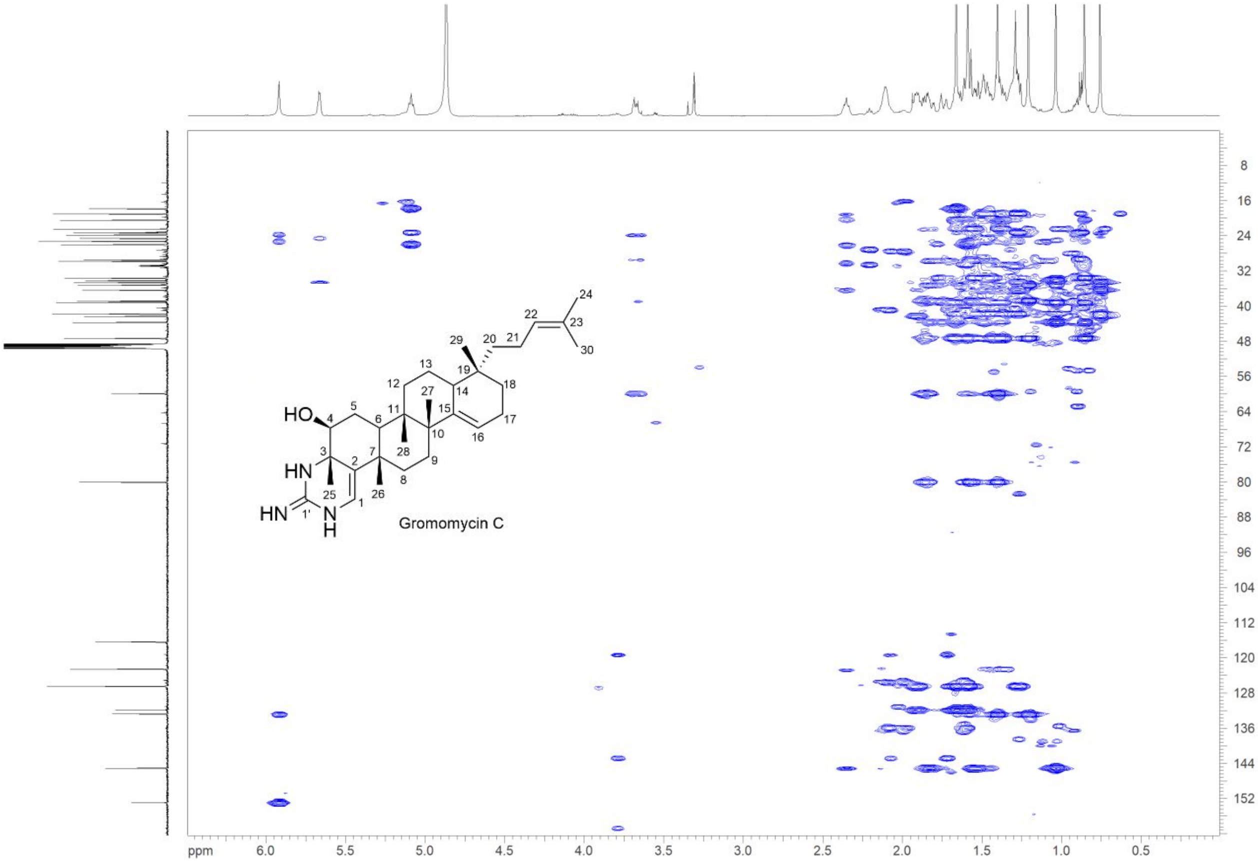

Figure S7: ^{1}H-NMR spectrum (500 MHz, $MeOD_4$) of gromomycin I (480A1).

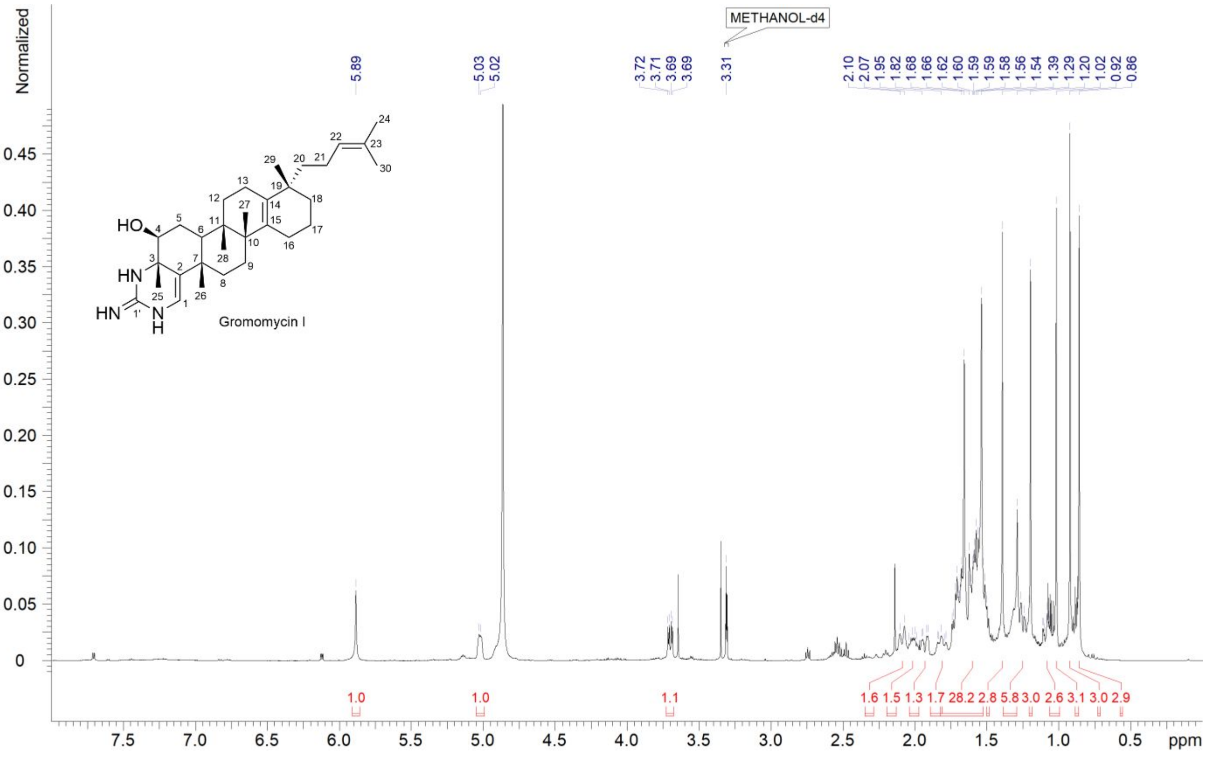

Figure S8: ^{13}C-NMR spectrum (125 MHz, $MeOD_4$) of gromomycin I (480A1).

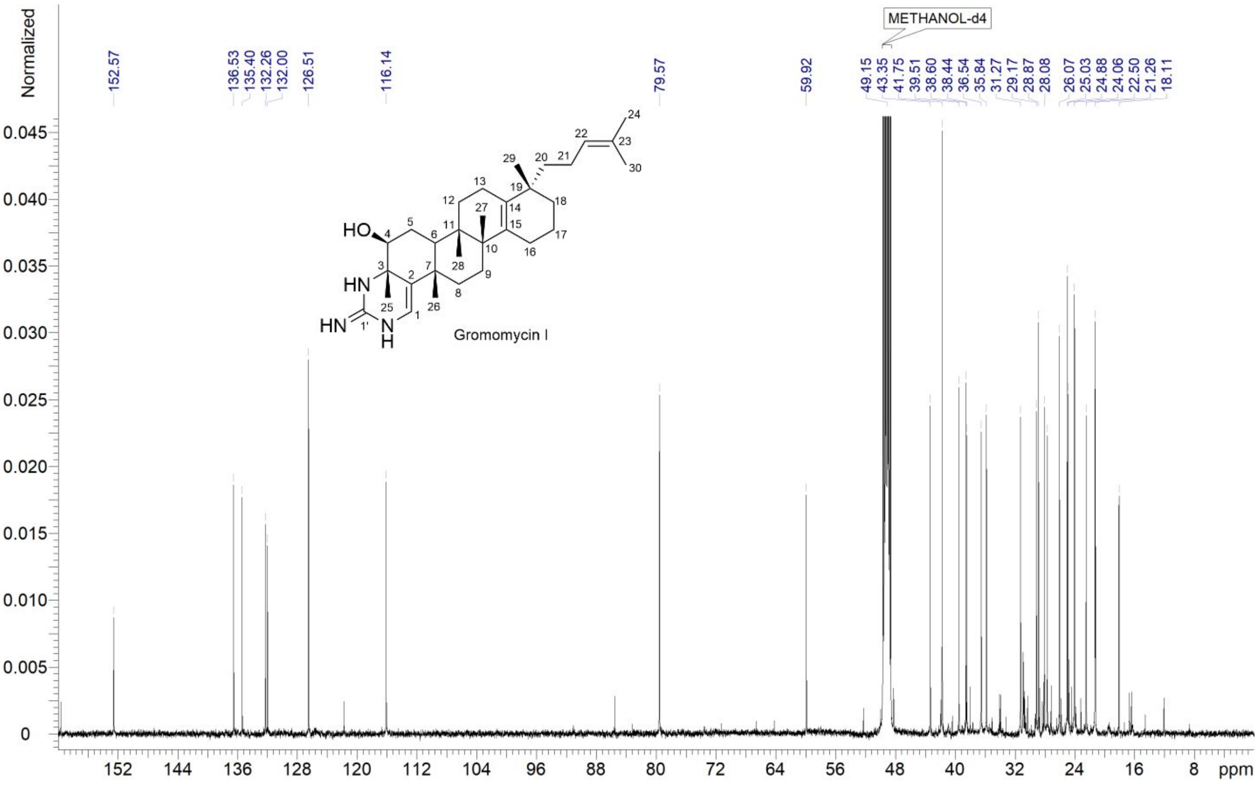

Figure S9: COSY spectrum (500 MHz, $MeOD_4$) of gromomycin I (480A1).

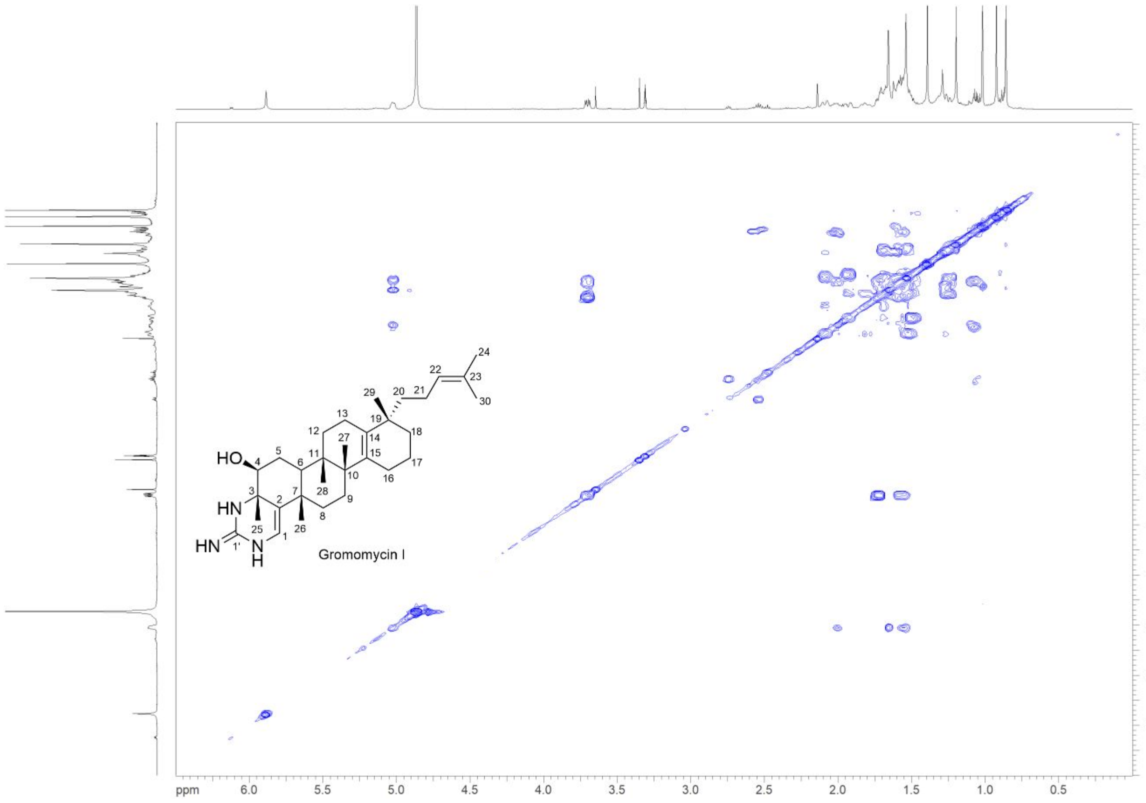

Figure S10: HSQC spectrum (MeOD$_4$) of gromomycin I (480A1).

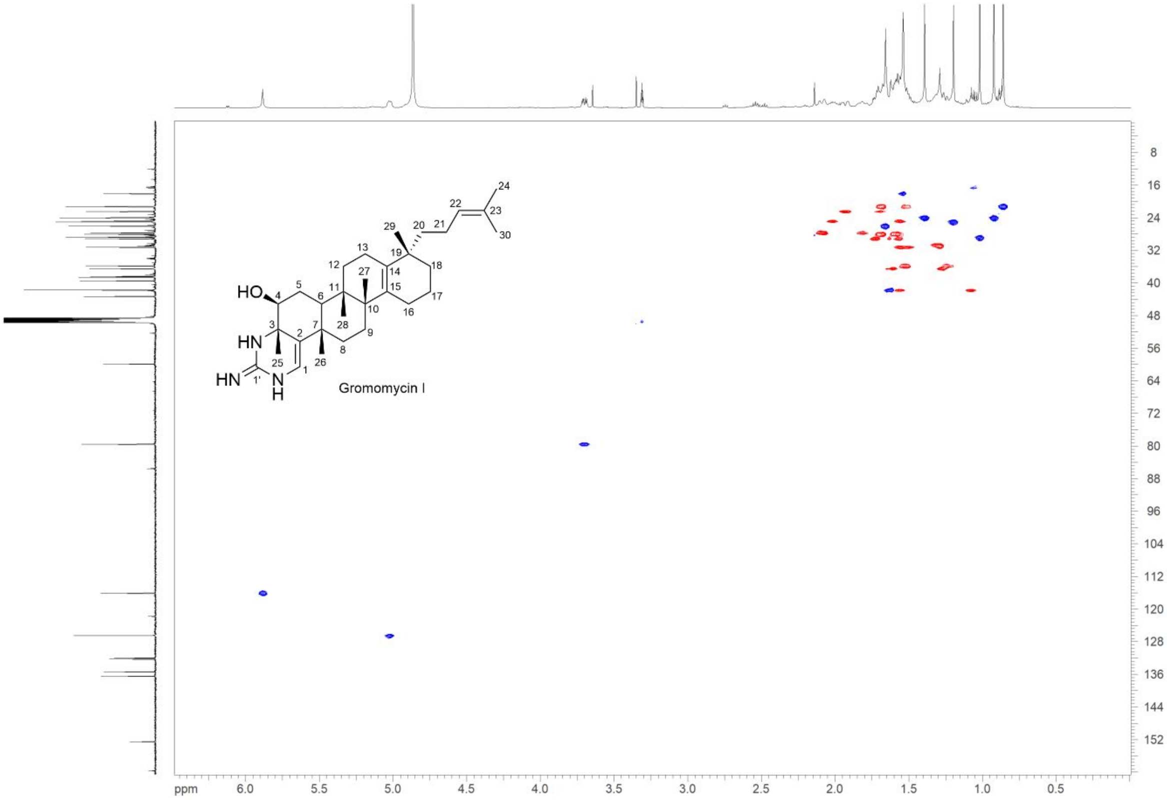

Figure S11: HMBC spectrum ($MeOD_4$) of gromomycin I (480A1).

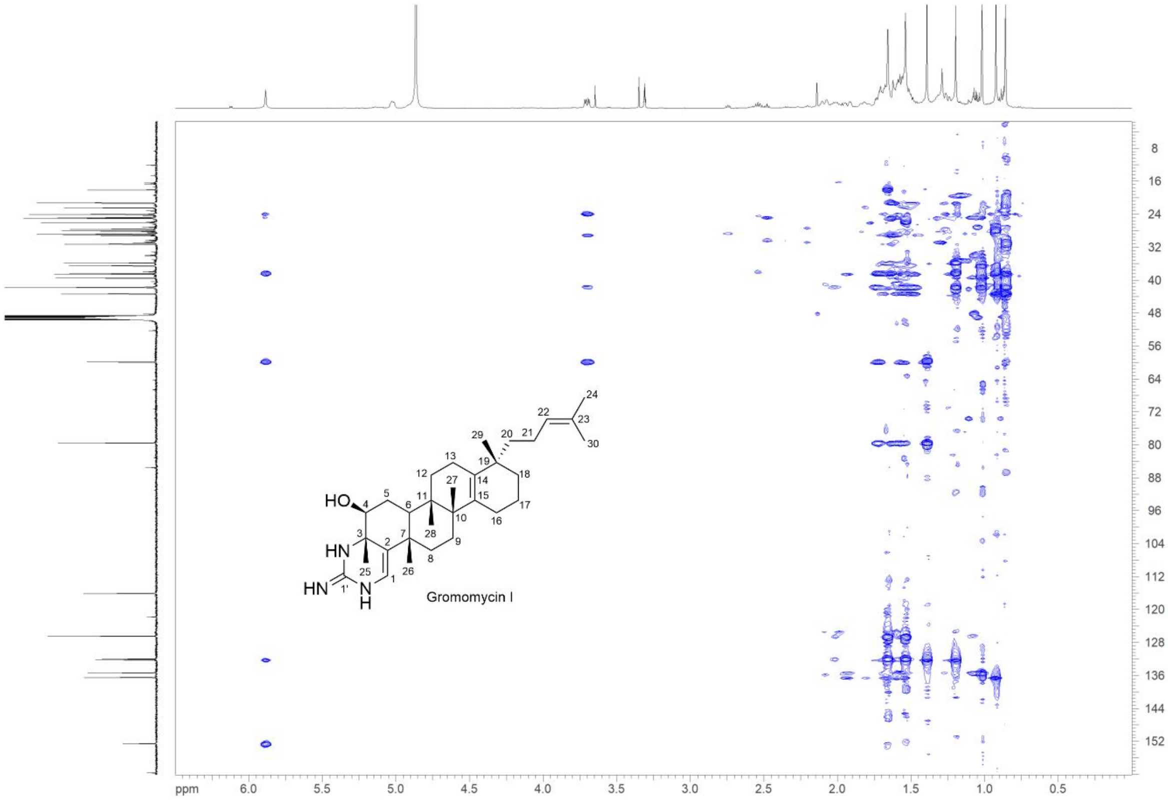

Figure S12: ^{1}H-NMR spectrum (500 MHz, MeOD₄) of gromomycin J (482A).

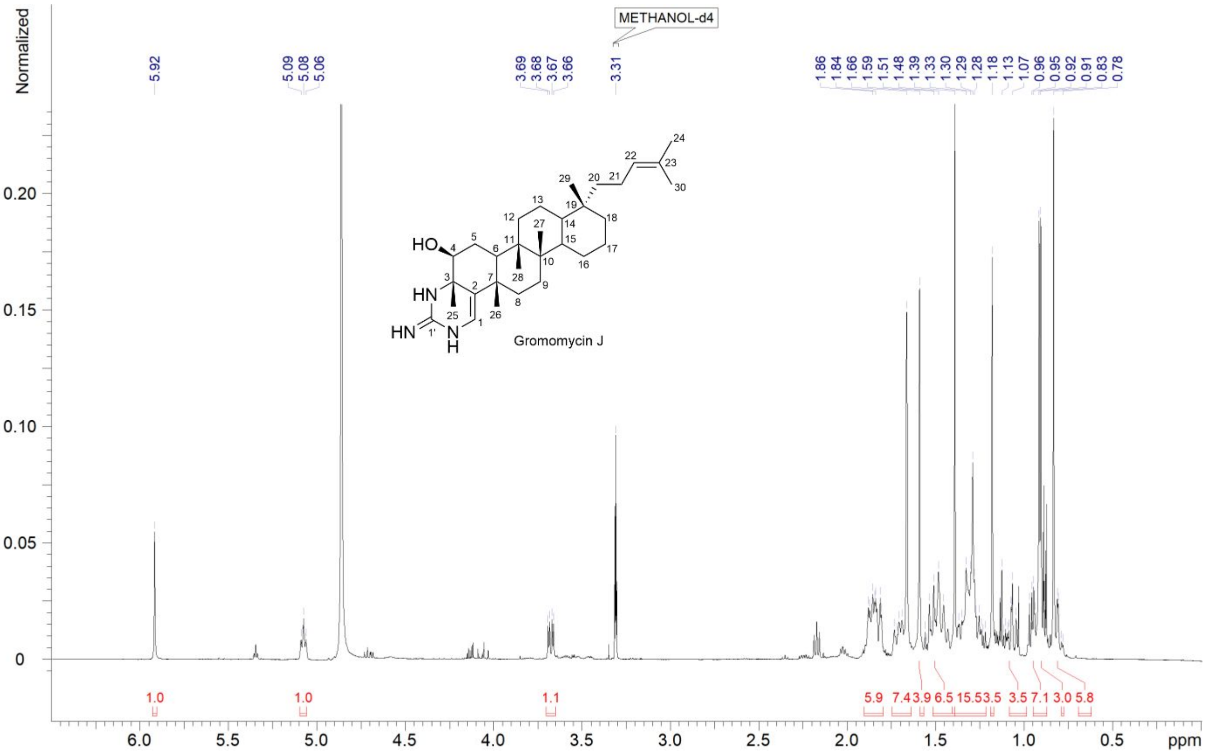

Figure S13: ^{13}C-NMR spectrum (125 MHz, $MeOD_4$) of gromomycin J (482A).

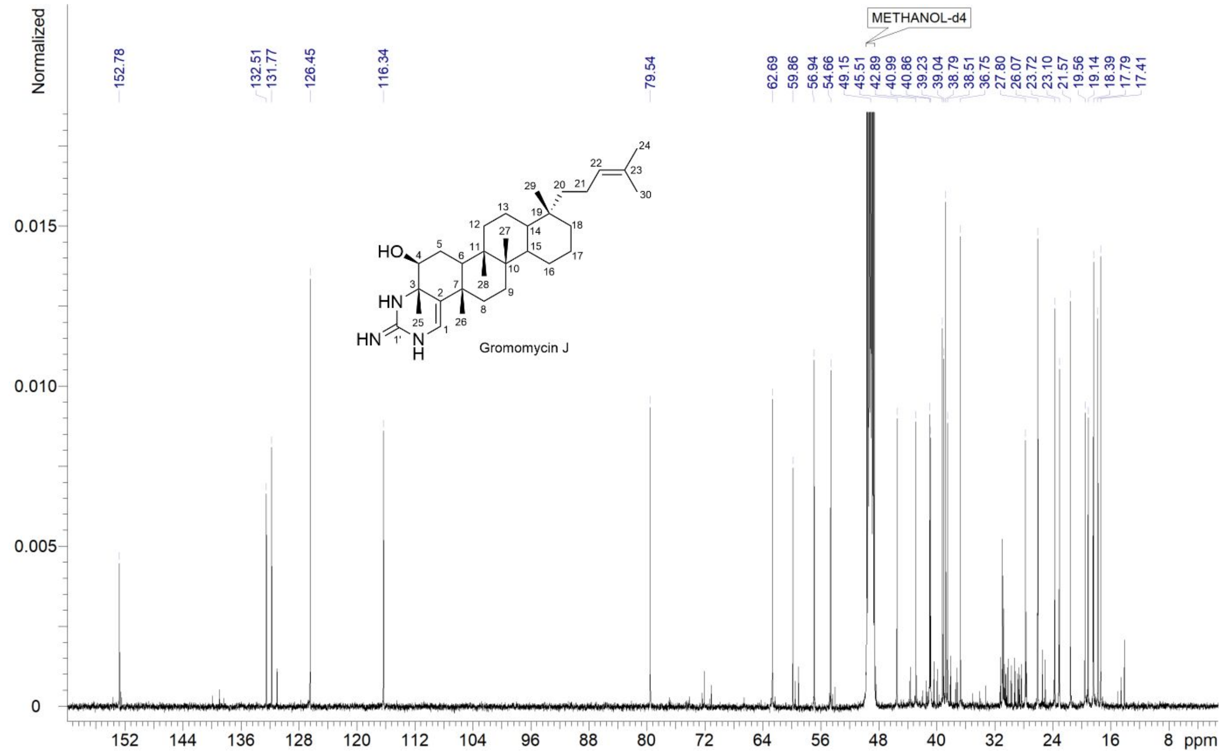

Figure S14: COSY spectrum (MeOD$_4$) of gromomycin J (482A).

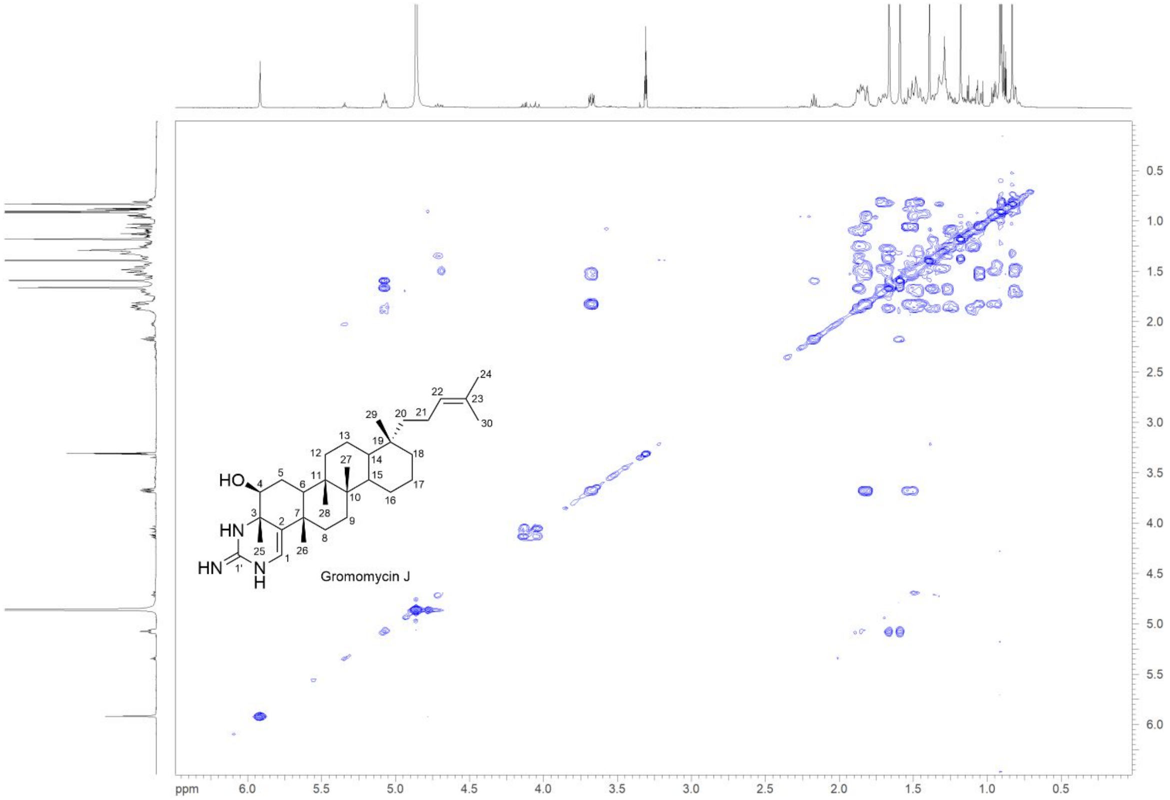

Figure S15: HSQC spectrum (MeOD$_4$) of gromomycin J (482A).

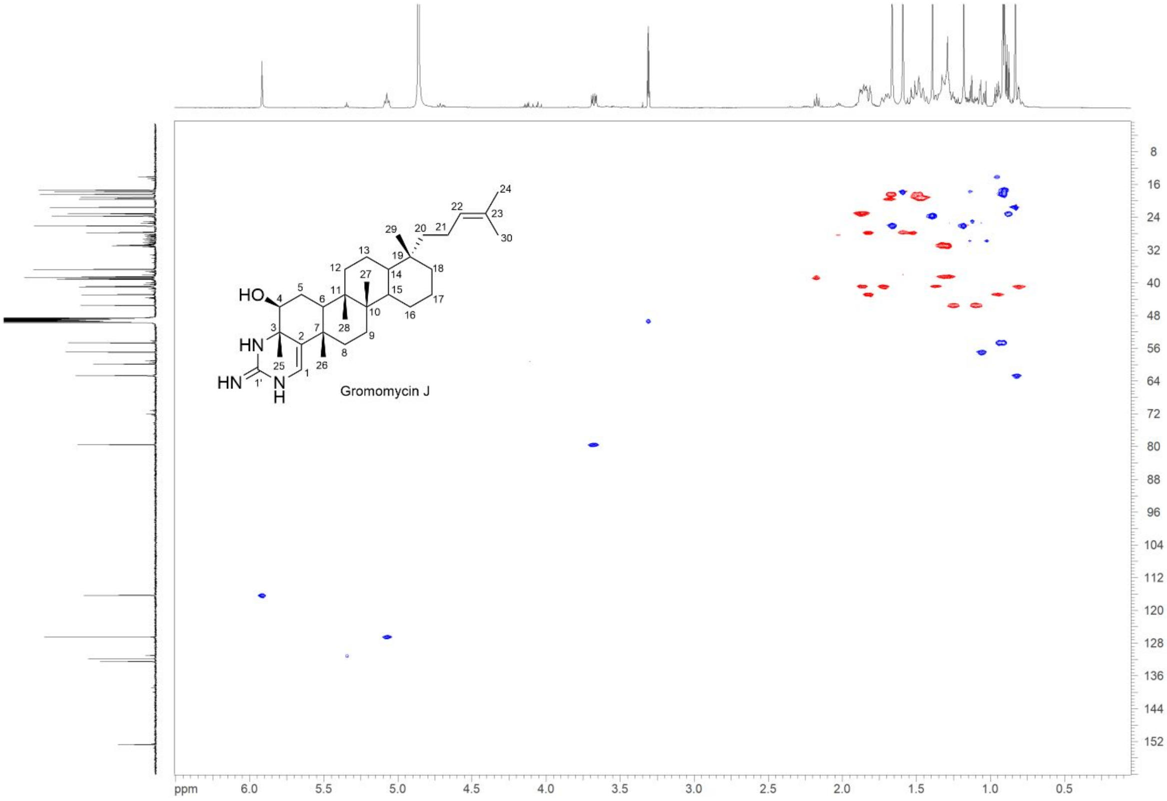

Figure S16: HMBC spectrum (MeOD$_4$) of gromomycin J (482A).

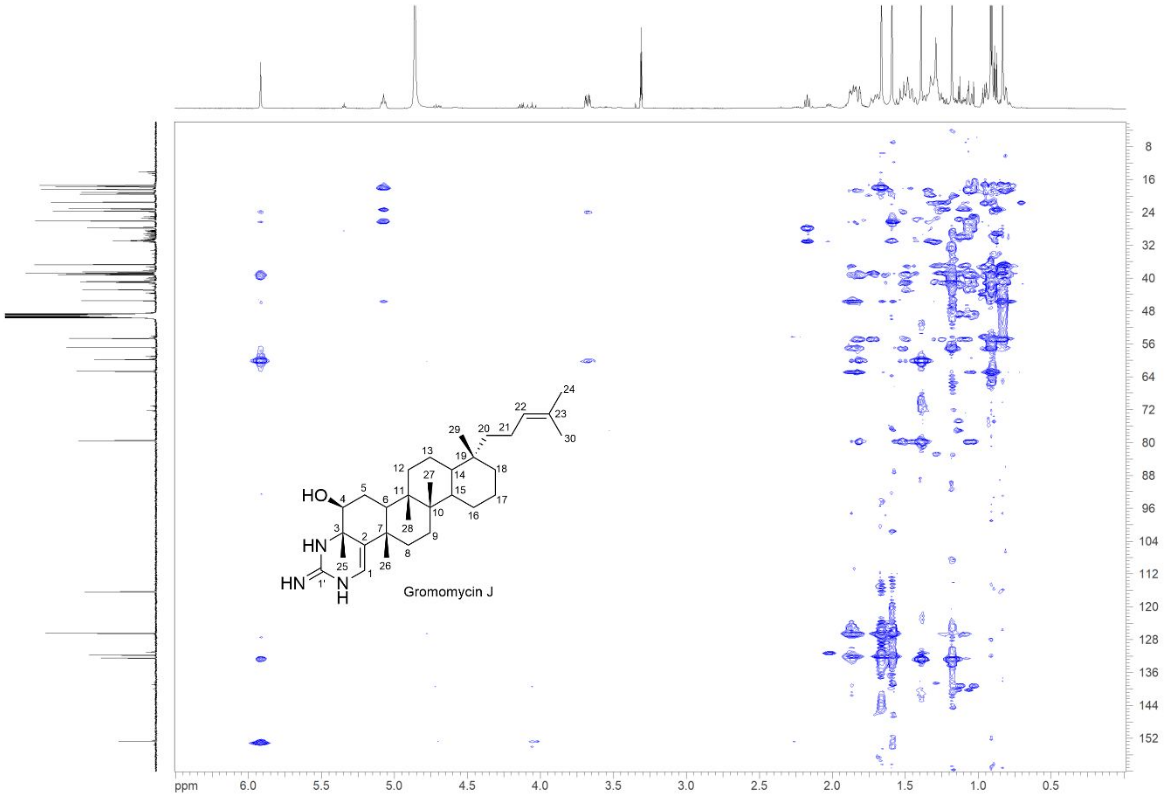

Figure S17: Bacterial phenotyping by scanning electron microscopy (SEM) imaging. SEM micrographs of *S. aureus* treated with 4- and 8-fold MIC of gromomycin I (T = 15 min). The control represents *S. aureus* cells exposed to DMSO. Scale bars are 300 nm. GRO, gromomycin I.

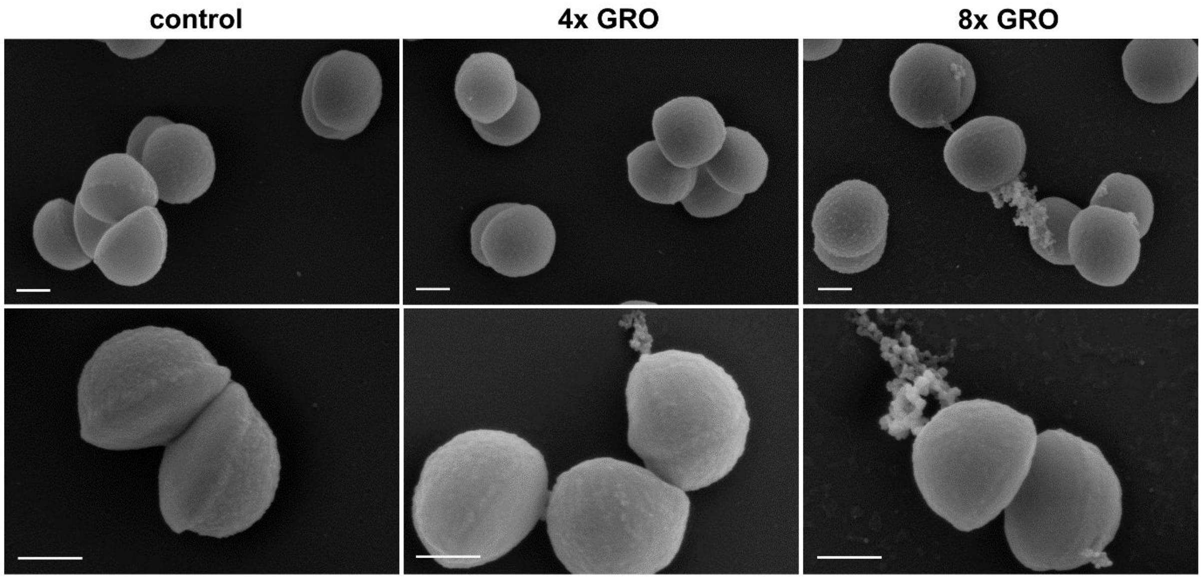

Figure S18: Gromomycin activity is impaired in the presence of fatty acids and phospholipids. A MIC values of gromomycin I against *S. aureus* ATCC29213 in the presence of different fatty acids and (phospho-) lipids. Lipids and fatty acids were used at 100 µM concentration. **B** Concentration dependency of activity neutralization (gromomycin: solid line, gentamicin: dashed line). GRO, gromomycin I.

A

Condition	MIC [µg/mL] GRO	MIC fold change
MHB2	2	-
+ DOPG (negative)	64	32
+ POPG (negative)	64	32
+ POPS (negative)	32	16
+ POPC (neutral)	16	8
+ POPE (neutral)	8	4
+ DOTAP (positive)	2	-
+ lauric acid (C12:0)	16	8
+ myristic acid (C14:0)	32	16
+ pentadecanoic acid (C15:0)	16	8
+ palmitic acid (C16:0)	8	4
+ palmitoleic acid (C16:1)	64	32
+ stearic acid (C18:0)	8	4
+ linoleic acid (C18:2)	64	32

B

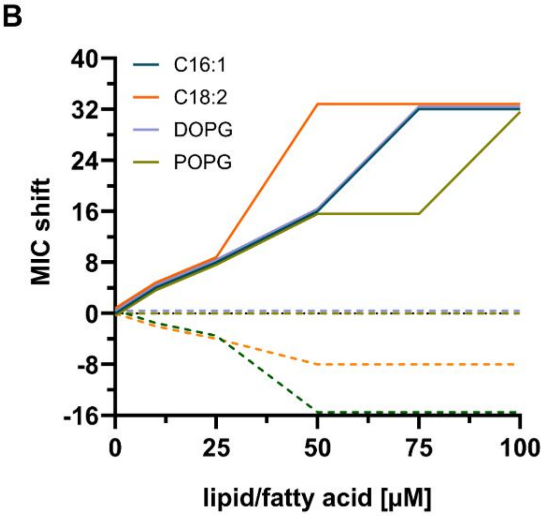

Figure S19. Mass spectra analysis (m/z) of Gromomycins I, C,

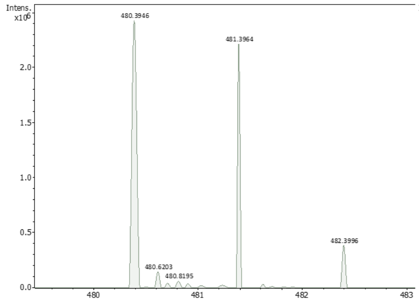

Gromomycin I (480A1)

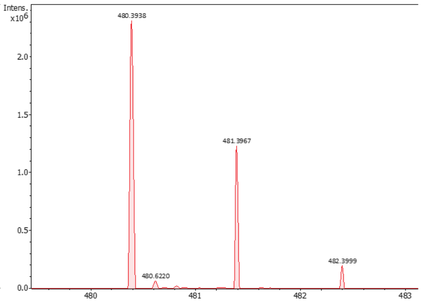

Gromomycin C (480A2)

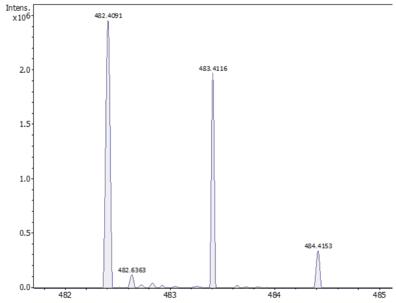

Gromomycin J (482A)

Figure S20: All *gro*BGCs identified with CluSeek tool.[5] The homologs of *groD*, *groF* and *groH* genes are highlighted in red.

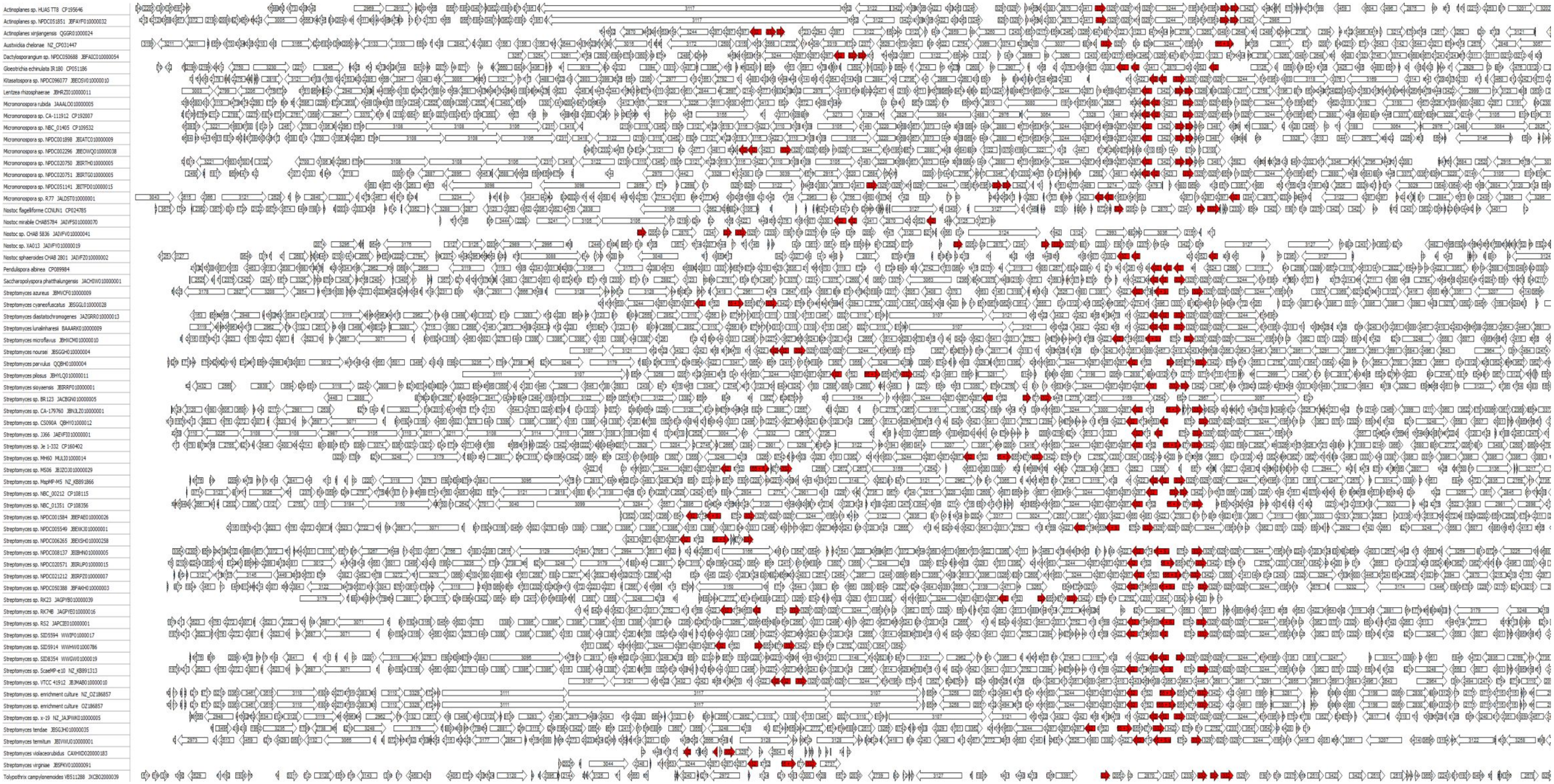

References

(1) Myronovskyi, M.; Rosenkränzer, B.; Nadmid, S.; Pujic, P.; Normand, P.; Luzhetskyy, A. Generation of a Cluster-Free *Streptomyces albus* Chassis Strains for Improved Heterologous Expression of Secondary Metabolite Clusters. *Metab. Eng.* **2018**, *49*, 316-324.

(2) Ahmed, Y.; Rebets, Y.; Estévez, M. R.; Zapp, J.; Myronovskyi, M.; Luzhetskyy, A. Engineering of *Streptomyces lividans* for Heterologous Expression of Secondary Metabolite Gene Clusters. *Microb. Cell Fact.* **2020**, *19*, 1-16.

(3) Flett, F.; Mersinias, V.; Smith, C. P. High efficiency intergeneric conjugal transfer of plasmid DNA from Escherichia coli to methyl DNA-restricting streptomycetes. *FEMS Microbiology Letters* **1997**, *155* (2), 223-229. DOI: https://doi.org/10.1111/j.1574-6968.1997.tb13882.x.

(4) Tistechok, S.; Bratiichuk, D.; Sucipto, H.; Gummerlich, N.; Stierhof, M.; Gromyko, O.; Fries, F.; Fedorenko, V.; Müller, R.; Zapp, J. Gromomycins: An Unprecedented Class of Triterpene Antibiotics Produced by a Novel Biosynthetic Pathway. *Angew. Chem. Int. Ed.* **2025**, *64* (22), e202422270.

(5) Hrebicek, O.; Kadlcik, S.; Najmanova, L.; Janata, J.; Kamanova, J.; Hanzlikova, L.; Koberska, M.; Kovarovic, V.; Kamenik, Z. CluSeek: Bioinformatics Tool to Identify and Analyze Gene Clusters. *bioRxiv* **2025**, 2025.2009.2016.676505.
